# The WAVE complex associates with sites of saddle membrane curvature

**DOI:** 10.1083/jcb.202003086

**Published:** 2021-06-07

**Authors:** Anne Pipathsouk, Rachel M. Brunetti, Jason P. Town, Brian R. Graziano, Artù Breuer, Patrina A. Pellett, Kyle Marchuk, Ngoc-Han T. Tran, Matthew F. Krummel, Dimitrios Stamou, Orion D. Weiner

**Affiliations:** 1 Cardiovascular Research Institute, University of California, San Francisco, San Francisco, CA; 2 Department of Biochemistry and Biophysics, University of California, San Francisco, San Francisco, CA; 3 Nano-Science Center and Department of Chemistry, University of Copenhagen, Copenhagen, Denmark; 4 GE Healthcare, Life Sciences, Cell Analysis Division, Marlborough, MA; 5 Department of Pathology and Biological Imaging Development CoLab, University of California, San Francisco, San Francisco, CA

## Abstract

How local interactions of actin regulators yield large-scale organization of cell shape and movement is not well understood. Here we investigate how the WAVE complex organizes sheet-like lamellipodia. Using super-resolution microscopy, we find that the WAVE complex forms actin-independent 230-nm-wide rings that localize to regions of saddle membrane curvature. This pattern of enrichment could explain several emergent cell behaviors, such as expanding and self-straightening lamellipodia and the ability of endothelial cells to recognize and seal transcellular holes. The WAVE complex recruits IRSp53 to sites of saddle curvature but does not depend on IRSp53 for its own localization. Although the WAVE complex stimulates actin nucleation via the Arp2/3 complex, sheet-like protrusions are still observed in ARP2-null, but not WAVE complex-null, cells. Therefore, the WAVE complex has additional roles in cell morphogenesis beyond Arp2/3 complex activation. Our work defines organizing principles of the WAVE complex lamellipodial template and suggests how feedback between cell shape and actin regulators instructs cell morphogenesis.

## Teaser

The local rules that control cell shape and movement are not understood. Here, Pipathsouk et al. discover a novel nanoscale organization of a key actin regulator that could explain how cellular protrusions expand and self-straighten and how transcellular holes are repaired.

## Introduction

Cells manipulate the shape of their plasma membranes to execute physiological functions ranging from building the protrusions that drive cell motility to coordinating the membrane deformations that enable endocytosis. Actin polymerization plays a major role in coordinating these processes, but how cells specify the proper pattern of actin assembly to achieve these distinct shapes is not known. Cells use nucleation promoting factors (NPFs) to spatially and temporally control their patterns of actin polymerization (Takenawa and Suetsugu, 2007; Chesarone and Goode, 2009). For example, neural Wiskott–Aldrich syndrome protein (N-WASP) and WASP-family verprolin homologous protein (WAVE) activate the actin-related protein 2/3 (Arp2/3) complex to seed actin nucleation (Machesky and Insall, 1998; Pollard and Borisy, 2003; Machesky et al., 1999).

**Figure 1.**
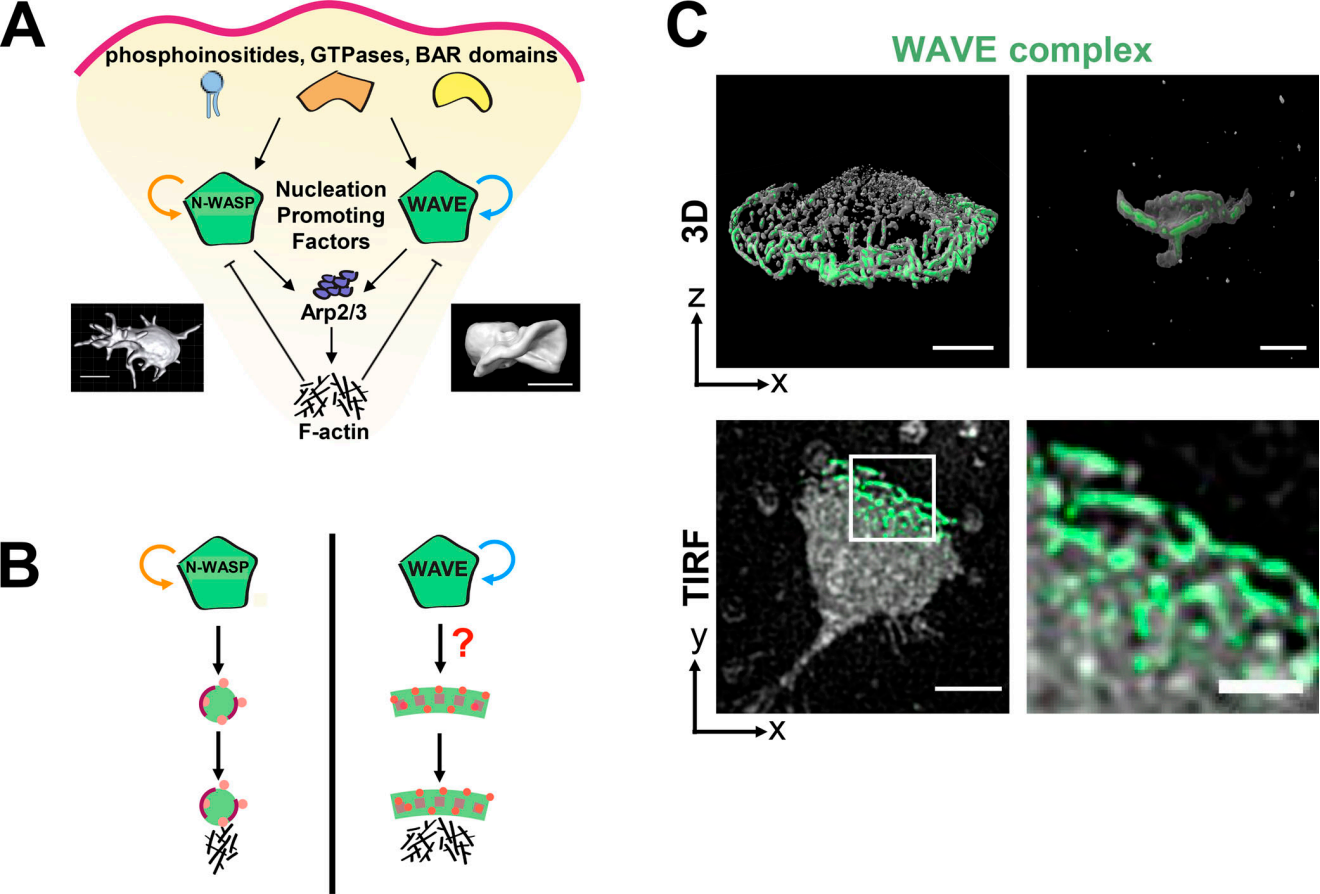
**How does the WAVE complex achieve the proper spatial organization for lamellipodial generation? (A)** Homologous actin NPFs organize different cell morphologies. Given that the homologous NPFs N-WASP and the WAVE complex are both activated by homologous GTPases, associated with homologous BAR domains, stimulated Arp2/3 complex activation, and stripped off the membrane as a function of this actin polymerization, how do they organize different cellular morphologies? N-WASP canonically associates with filopodia, invadopodia, and endocytosis, whereas the WAVE complex associates with sheet-like lamellipodia. Left: Chick cranial neural crest cell with multiple filopodia from Genuth et al. (2018). Right: Head-on view of a neutrophil-like dHL60 lamellipodium (ChimeraX rendering of a confocal z-stack). Scale bars: 10 µm (left) and 5 µm (right). **(B)** Schematic of how NPF spatial organization could instruct the resulting actin morphologies. N-WASP’s positive feedback results in the focal organization expected for one-dimensional, finger-like actin structures (Banjade and Rosen, 2014). To build lamellipodia, does the WAVE complex’s positive feedback result in a linear organization to template 2D, sheet-like actin structures? **(C)** Hem1-EGFP, a fluorescently tagged subunit of the WAVE complex, has a linear organization at tips of lamellipodia in chemoattractant-stimulated (10 nM fMLP) dHL60 cells. Top: 3D imaging of the WAVE complex at tips of extending lamellipodia; left, widefield 3D reconstruction; right, lattice light sheet reconstruction of ruffles from a head-on view. Bottom: WAVE complex’s linear organization viewed from the ventral plasma membrane; simultaneous TIRF imaging of Hem1-EGFP (green) and membrane CellMask DeepRed dye (gray). Scale bars: 5 µm and 2 µm (bottom right). See Video 1.

N-WASP and WAVE are nested in similar signaling topologies. Both are stimulated by phosphoinositides, Rho GTPases, and curvature-sensitive Bin-amphiphysin-Rvs (BAR) domain proteins; both activate the Arp2/3 complex; both are recycled in an actin-dependent fashion; and both show evidence of oligomerization at the membrane (Fig. 1 A; Takenawa and Suetsugu, 2007; Pollard and Borisy, 2003; Rohatgi et al., 1999; Oikawa et al., 2004; Weiner et al., 2007; Ho et al., 2004; Koronakis et al., 2011; Suetsugu et al., 2006; Abou-Kheir et al., 2008; Etienne-Manneville and Hall, 2002). Despite these similarities, the morphological structures they build are distinct: N-WASP typically participates in spiky filopodial protrusions, invadopodia, and endocytic vesicles (Takenawa and Suetsugu, 2007; Miki et al., 1998; Bu et al., 2009; Naqvi et al., 1998; Yamaguchi et al., 2005; Yu and Machesky, 2012; Yu et al., 2012; Benesch et al., 2005; Taylor et al., 2011; Veltman et al., 2012), whereas WAVE participates in broad, sheet-like lamellipodial protrusions (Hall, 1998; Weiner et al., 2006; Leithner et al., 2016; Graziano et al., 2019). Though these are their typical actin structures, WASP can compensate for the lack of Scar/WAVE in building lamellipodia in some cell types like *Dictyostelium discoideum* (Veltman et al., 2012; Zhu et al., 2016), but not in neutrophils or dendritic cells (Graziano et al., 2019; Leithner et al., 2016). The rules for instructing filopodial versus lamellipodial cell morphologies are not understood.

Biochemical reconstitutions have provided insights into how different patterns of actin nucleation lead to different morphological structures. Activation of the Arp2/3 complex in the absence of membranes produces a branched dendritic morphology (Mullins et al., 1998). Activation of the ARP2/3 complex on the surface of giant unilamellar vesicles produces filopodial-like structures (Liu et al., 2008). In contrast, spatially organizing Arp2/3 complex activation in a linear geometry via glass rods (Carlier et al., 2003) or UV micropatterned surfaces (Boujemaa-Paterski et al., 2017) produces lamellipodial-like structures. These data suggest that while spiky, finger-like actin structures are the default morphology for the Arp2/3 complex activation on membranes, lamellipodium formation requires a linear structural template of actin nucleators (Fritz-Laylin et al., 2017b). What forms the basis of the linear template for lamellipodia in living cells is not known.

In cells, N-WASP forms the focal structure expected for finger-like protrusions (Yu et al., 2012; Benesch et al., 2005; Case et al., 2019), and the WAVE complex propagates as linear waves at the edges of lamellipodia (Weiner et al., 2007; Fritz-Laylin et al., 2017a; Steffen et al., 2004; Davidson et al., 2018; Hahne et al., 2001; Huang et al., 2013; Zallen et al., 2002; Leithner et al., 2016). In the case of N-WASP, biochemical reconstitutions recapitulate its in vivo distribution: N-WASP and its multivalent binding partners generate biomolecular condensates (Case et al., 2019; Banjade and Rosen, 2014) that result in focal accumulation of the nucleator. In the presence of the Arp2/3 complex and actin, these puncta-shaped condensates promote focal bursts of F-actin that produce spiky protrusions (Banjade and Rosen, 2014). In contrast, we do not know the basis of the WAVE complex’s linear organization in cells (Fig. 1 B).

The WAVE complex is required for proper cell migration and regulation of cell shape across eukaryotes including mammals, amoeba, and plants (Pollard and Borisy, 2003; Weiner et al., 2007; Leithner et al., 2016; Fritz-Laylin et al., 2017a; Ibarra et al., 2006; Szymanski, 2005; Rakeman and Anderson, 2006; Kunda et al., 2003). As a pentameric heterocomplex, the WAVE complex contains the subunits WAVE/Scar, Abi1/2, HSPC300, Sra1/Cyfip1, and Nap1/Hem1 (Weiner et al., 2006; Eden et al., 2002; Innocenti et al., 2004). In neutrophils and other motile cells, the dynamics of the WAVE complex closely correspond to the leading edge’s morphology and pattern of advance (Weiner et al., 2007; Steffen et al., 2004; Davidson et al., 2018; Hahne et al., 2001; Huang et al., 2013; Zallen et al., 2002; Leithner et al., 2016; Fritz-Laylin et al., 2017a). The WAVE complex is required for the formation of lamellipodia and efficient chemotaxis in a range of immune cells (Weiner et al., 2006; Leithner et al., 2016; Graziano et al., 2019; Park et al., 2008; Evans et al., 2013; Kheir et al., 2005).

Here, we investigate the features of WAVE complex organization that may instruct the generation and dynamics of lamellipodia. Using super-resolution microscopy, we find that the WAVE complex forms actin-independent 230-nm-wide rings that localize to regions of saddle membrane curvature. To identify a potential mechanism of membrane curvature recognition, we explored the WAVE complex’s partnering interactions with insulin receptor tyrosine kinase substrate protein of 53 kD (IRSp53), an inverse BAR (I-BAR) domain protein, and found that IRSp53 depends on its interactions with the WAVE complex to recognize lamellipodial and saddle curvature geometries, but the WAVE complex can localize to these structures without IRSp53. While WAVE complex–null cells fail to form sheet-like protrusions, Arp2/3-disrupted cells can do so, highlighting the central role of the WAVE complex in this process. We propose that the WAVE complex’s nanoscale organization could explain emergent behaviors of cell morphogenesis including expanding and self-straightening lamellipodia and the recognition and closure of transcellular holes.

## Results

### The WAVE complex forms nanoscale, oligomeric rings in the absence of actin polymer

**Figure 2.**
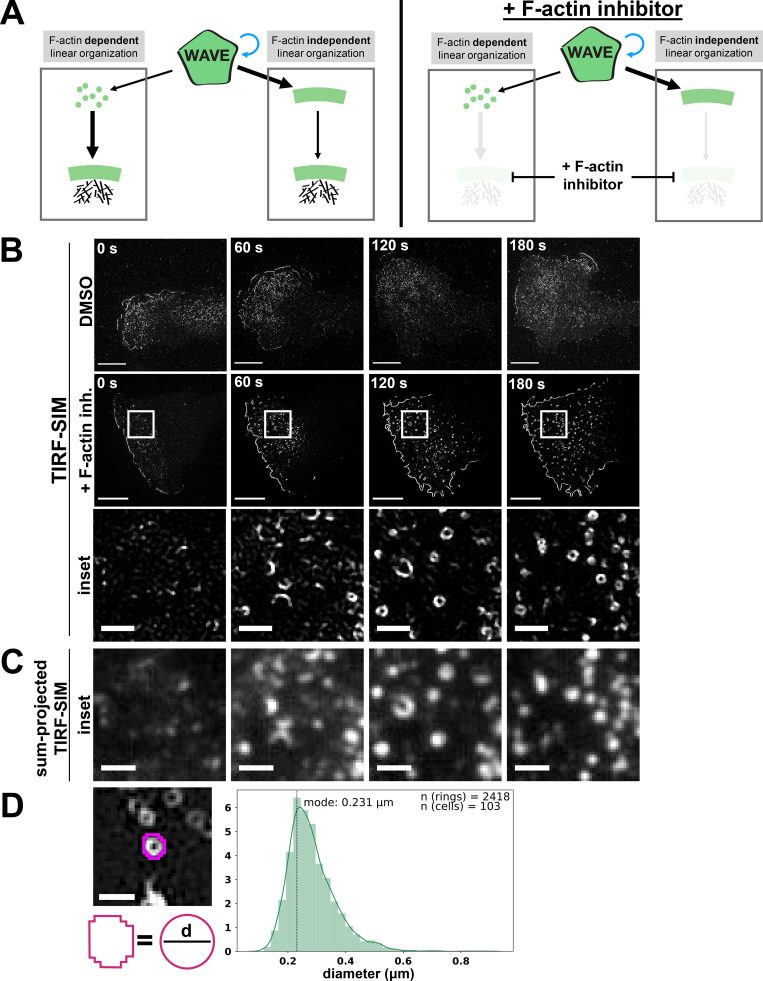
**WAVE complex forms 230-nm rings in the absence of the actin cytoskeleton. (A)** Models of how the WAVE complex could achieve its linear organization. Left: The WAVE complex could achieve its linear organization at the lamellipodial edge in a manner that is dependent on its interactions with the actin cytoskeleton (left) or that is established independently of the actin cytoskeleton (right). Right: Addition of F-actin inhibitor can be used to distinguish these models. **(B)** Super-resolution TIRF-SIM imaging reveals that the WAVE complex forms rings in the absence of actin polymer. dHL60 cells expressing Hem1-EGFP were acutely stimulated with DMSO (top row) or F-actin inhibitor (+ F-actin inh.; 5 µM latrunculin A final, middle and bottom rows) at 0 s. Bottom row shows the white boxed insets. Scale bars: 5 µm (top) and 1 µm (insets). See Video 2.** (C)** Conventional TIRF resolution comparison highlights the need for super-resolution microscopy to resolve diffraction-limited WAVE complex puncta as rings. Conventional resolution TIRF images were created by sum-projecting the nine images (three phases × three angles) that construct the TIRF-SIM images of B. Scale bar: 1 µm. See Video 2.** (D)** The WAVE complex forms stereotypical 230 nanometer sized rings. Left, example measurement of fitting the ring’s perimeter to a perfect circle and calculating the circle’s diameter, d. Scale bar: 500 nm. Right, histogram of diameters of rings across a range of F-actin inhibiting drugs and concentrations, *n* = 2,418 rings from 103 cells, mode = 230 nm, median = 270 nm (see Fig. S2, A and B).

In neutrophil-like differentiated HL60 (dHL60) cells and a range of other motile cells, the WAVE complex can be seen propagating as a linear “wave” structure at the edges of membrane ruffles and lamellipodia (Fig. 1 C and Video 1; Oikawa et al., 2004; Weiner et al., 2007; Veltman et al., 2012; Leithner et al., 2016; Rakeman and Anderson, 2006). This dynamic propagation arises from an excitable feedback network with positive feedback (WAVE complex recruits more WAVE complex) and negative feedback loops (WAVE complex stimulates actin polymerization, which inhibits WAVE complex’s association with the membrane; Weiner et al., 2007; Millius et al., 2012). One source of negative feedback is the force of actin polymerization that strips the WAVE complex off the plasma membrane (Millius et al., 2012). In contrast, the mechanism and spatial organization of positive feedback, i.e., WAVE complex recruiting more WAVE complex, are not well understood. We were particularly interested in understanding whether the linear patterns of the WAVE complex in lamellipodia are dependent on an interaction between these positive and negative feedback loops, as is the case for other excitable networks (Allard and Mogilner, 2013), or whether the WAVE complex’s oligomerization at the plasma membrane has a specific geometric organization in the absence of the actin-based negative feedback loop (Fig. 2 A). We can use inhibitors of actin polymerization to deplete F-actin to distinguish between the two models.

**Video 1.** **Lattice light sheet and TIRF imaging of WAVE complex at protruding lamellipodia.** Left: dHL60 cell expressing Hem1-EGFP (green) continually exposed to chemoattractant (25 nM fMLP) imaged with lattice light sheet at one frame every 4 s. Gray overlay represents the cell boundary defined by cytosolic Hem1. This video corresponds to Fig. 1 C, top right. Right: dHL60 cell expressing Hem1-EGFP (green) and stained with membrane dye (CellMask DeepRed; gray) continually exposed to chemoattractant (10 nM fMLP). Both channels were imaged simultaneously with TIRF at one frame every 2 s. Scale bar: 5 µm. This video corresponds to Fig. 1 C, bottom.

**Figure S1.**
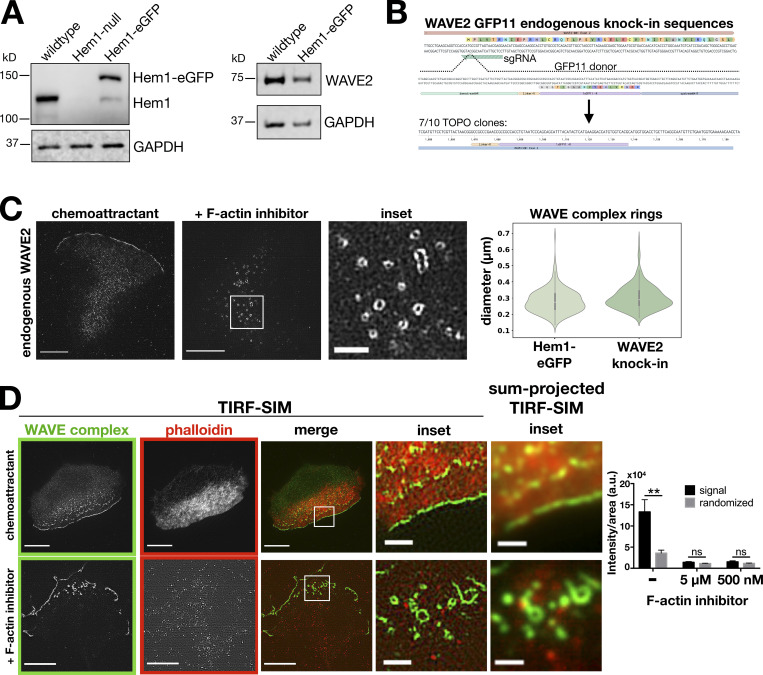
**WAVE complex rings are not due to overexpression of WAVE complex subunits and are devoid of F-actin.(A)** HEM1 and WAVE2 expression levels in the Hem1-EGFP HL60 cell line used throughout the study. Left: Western blot of HEM1 and GAPDH in wild type, HEM1-null, and Hem1-EGFP HL60 cell lines. Right: Western blot of WAVE2 and GAPDH in wild type and Hem1-EGFP HL60 cell lines. **(B)** WAVE2 split-GFP endogenous knock-in sequences. In a GFP1-10–expressing cell line, CRISPR/Cas9 cuts the N terminus of WAVE2’s exon 2 and the cell repairs with a GFP11 donor flanked with WAVE2 homology arms. **(C)** Endogenous localization of WAVE2 in split-GFP knock-in dHL60 cells. Cells treated with chemoattractant (10 nM fMLP) or F-actin inhibitor (500 nM latrunculin B). TIRF-SIM imaging; scale bars: 5 µm and 1 µm (inset). Violin plot of the WAVE complex ring diameters in Hem1-EGFP and WAVE2 knock-in cells treated with F-actin inhibitor (500 nM latrunculin B); Hem1-EGFP *n* = 293 rings from 13 cells, WAVE2 knock-in *n* = 305 from 10 cells; cells pooled from at least two independent experiments per condition. Diameters measured in the same fashion shown in Fig. 2 D.** (D)** Images of dHL60 cells expressing Hem1-EGFP (green) treated with chemoattractant (100 nM fMLP; top) or F-actin inhibitor (5 µM latrunculin B; bottom) that were then fixed and stained with phalloidin (red). TIRF-SIM imaging; scale bars: 5 µm and 1 µm (insets). Convention resolution TIRF inset images are the sum-projection of the nine images (three phases × three angles) that construct TIRF-SIM images. Graph shows mean ± SEM of phalloidin intensity/area per cell; (−) F-actin inhibitor *n* = 14 cells, 5 µM latrunculin B F-actin inhibitor *n* = 10 cells, 500 nM latrunculin B F-actin inhibitor *n* = 10 cells; cells pooled from at least three independent experiments; multiple *t* tests comparing the phalloidin staining (signal) and a scrambled version of the phalloidin image (randomized); **, P = 0.003 < 0.01; ns, not significant; 5 µM F-actin inhibitor, P = 0.14 > 0.05; and 500 nM F-actin inhibitor, P = 0.17 > 0.05.

When we previously visualized the pattern of the WAVE complex organization (via fluorescently tagged hematopoietic protein 1 [Hem1], a subunit of the WAVE complex) in the absence of the actin cytoskeleton, standard total internal reflection fluorescence (TIRF) microscopy showed amorphous punctate structures of the WAVE complex (Weiner et al., 2007). For our current study, we revisited this experiment with super-resolution microscopy, specifically TIRF–structured-illumination microscopy (TIRF-SIM), a technique that enables a twofold increase in resolution in the TIRF plane. When the F-actin inhibitor latrunculin A was acutely added to dHL60 cells to deplete F-actin, TIRF-SIM imaging of Hem1-EGFP revealed that the WAVE complex organizes into highly stereotyped 230-nm-wide ring structures (Fig. 2, B–D; and Video 2). Importantly, super-resolution microscopy was required to resolve the nanometer-scale rings because they otherwise appear as amorphous blob-like structures by conventional TIRF (Fig. 2 C). The WAVE complex rings were not an artifact of overexpressing a tagged subunit, as any subunit protein not incorporated into the multiprotein complex gets degraded (Fig. S1 A; Kunda et al., 2003; Weiner et al., 2006; Litschko et al., 2017; Graziano et al., 2019). Moreover, endogenously labeled WAVE2 also formed rings upon F-actin inhibition (Fig. S1, B and C). Furthermore, the WAVE complex rings are devoid of F-actin: phalloidin staining of cells treated with latrunculin shows that the rings lack detectable residual filamentous actin (Fig. S1 D).

**Video 2.** **TIRF-SIM versus sum-projected TIRF-SIM imaging of WAVE complex rings in the absence of actin polymer**. dHL60 cell expressing Hem1-EGFP continually exposed to chemoattractant (10 nM fMLP) and acutely treated with latrunculin A (5 µM) at 30 s. To create a conventional resolution (~TIRF) comparison to highlight the requirement of super-resolution microscopy to resolve diffraction-limited WAVE complex puncta as rings, the nine images (three phases × three angles) that construct TIRF-SIM images were sum-projected. TIRF-SIM (left) versus ~TIRF (right) imaging, one frame every 4 s. Scale bar: 5 µm. This video corresponds to Fig. 2, B and C.

**Figure S2.**
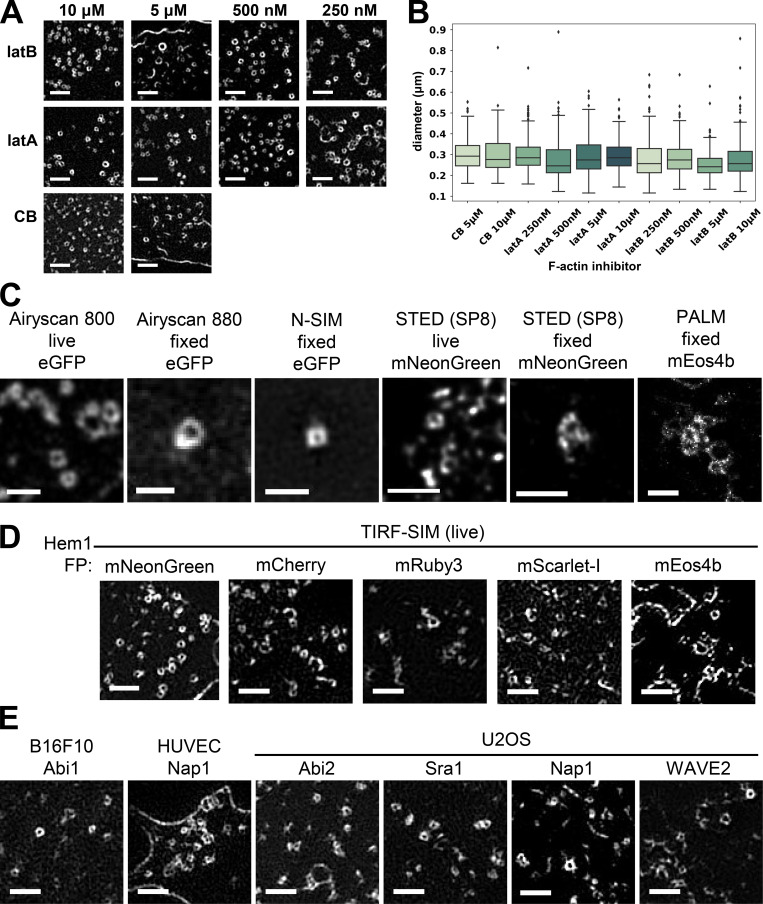
**WAVE complex rings observed for multiple cell lines, labeled subunits, microscopy techniques, and actin inhibitors.(A)** Zoomed-in TIRF-SIM images of Hem1-EGFP rings across different drug conditions: 250 nM, 500 nM, 5 µM, and 10 µM (final) of latrunculin B (latB), latrunculin A (latA), and cytochalasin B (CB). Scale bar: 1 µm. **(B)** Boxplots (interquartile range) of ring diameters across drug conditions as measured in Fig. 2 D. Each condition has *n* = 10 cells except for latB 500 nM, which has *n* = 13 cells, and all cells are from at least three independent experiments per condition; ensemble histogram in Fig. 2 D. **(C)** Multiple super-resolution techniques in live or fixed cell conditions of Hem1 tagged with different fluorescent proteins all show ring structures. Microscopes and techniques used Airyscan 800 (Zeiss), Airyscan 880 (Zeiss), N-SIM (Nikon), SP8 STimulated Emission Depletion (STED; Leica), and photoactivated localization microscopy (PALM; B. Huang laboratory [UCSF] microscope). All cells treated with latrunculin B (500 nM). Scale bar: 1 µm. **(D)** Hem1 tagged with different fluorescent proteins (FP) show ring structures. All imaged with TIRF-SIM and treated with latrunculin B (500 nM). Scale bar: 1 µm. **(E)** Different EGFP-tagged WAVE complex subunits in B16F10 *(Mus musculus* skin melanoma cells), HUVECs, and U2OS (*Homo sapiens* bone osteosarcoma) cell lines show ring structures. All cells treated with latrunculin B (500 nM). TIRF-SIM imaging; scale bar: 1 µm.

The WAVE complex rings were observed across a range of F-actin inhibitors and concentrations (Fig. S2, A and B), super-resolution modalities (Fig. S2 C), tagged fluorescent proteins (Fig. S2 D), cell types, and specific WAVE complex subunits (Fig. S2 E). The rings may represent the favored oligomeric organization of the WAVE complex when freed from the constraints of the cytoskeleton and/or tension in the plasma membrane (Diz-Muñoz et al., 2016; Lieber et al., 2013). These experiments suggest that the WAVE complex forms nanoscale, F-actin–free, oligomeric rings at the plasma membrane.

### The formation of WAVE complex rings is dependent on upstream signals

**Figure 3.**
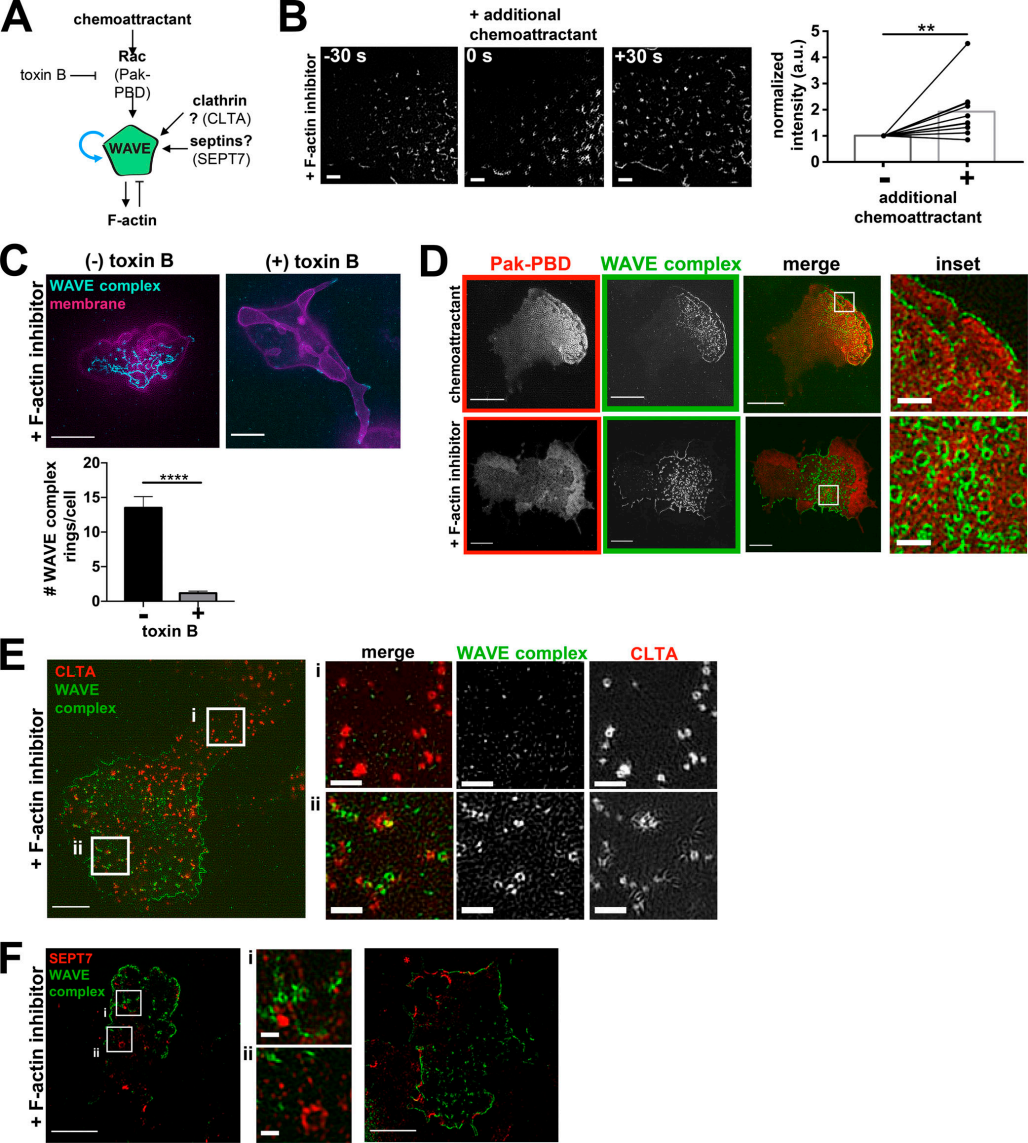
**The formation of WAVE complex rings is dependent on upstream signals. (A)** Potential inputs into the formation of WAVE complex rings. **(B)** WAVE complex rings form upon stimulation of additional chemoattractant. Additional WAVE complex (Hem1-EGFP) is recruited to the plasma membrane upon additional chemoattractant stimulation (100 nM fMLP, time point 0 s) in F-actin–inhibited (500 nM latrunculin B) cells. TIRF-SIM imaging; scale bar: 1 µm. Graph shows normalized integrated intensities of the WAVE complex before (−) and after (+) additional chemoattractant stimulation of F-actin–inhibited cells; *n* = 10 cells pooled from at least three independent experiments of TIRF-SIM imaging; paired *t* test, two-tailed; **, P = 0.0053 < 0.01. **(C)** dHL60s expressing WAVE complex (Hem1-EGFP; cyan) and membrane (CAAX-tagBFP; magenta) treated with (+) or without (−) *C. difficile* toxin B for at least 4 h and acutely treated with latrunculin B (500 nM final). Scale bar: 5 µm. Graph comparing the mean ± SEM number of the WAVE complex rings in cells treated with (+) and without (−) toxin B; (−) toxin B, *n* = 37 cells; (+) toxin B, *n* = 66 cells; cells pooled from the same three independent experiments; unpaired *t* test, two-tailed; ****, P < 0.0001. **(D)** Colocalization of Pak-PBD-mCherry (red) and Hem1-EGFP (green) in dHL60s treated with chemoattractant (10 nM fMLP final; top) and latrunculin B (500 nM final; bottom). Median filter applied to inset images. Scale bars: 5 µm and 1 µm (inset). **(E)** Distribution of endogenously labeled split-mNeonGreen clathrin light chain A (CLTA; red) and Hem1-mCherry (green) dHL60s treated with latrunculin B (500 nM final). Insets (i and ii) shown on right. Scale bars: 5 µm and 1 µm (insets). **(F)** Distribution of SEPT7 (immunofluorescence; red) in Hem1-EGFP (green) dHL60 cell line treated with latrunculin B (500 nM final). Left image shows WAVE complex rings (inset i) and septin rings (inset ii). Right image shows complementary localization of Hem1 and SEPT7 along the cell periphery. Scale bars: 5 µm and 0.5 µm (insets).

While WAVE complex activation is dependent on upstream regulators (Lebensohn and Kirschner, 2009; Leng et al., 2005), it was unclear whether these activators are also required for its formation of nanoscale rings (Fig. 3 A). We found that stimulation of F-actin–inhibited cells with chemoattractant increased the membrane-bound WAVE complex signal, indicating facilitation by chemoattractant receptor-mediated activation (Fig. 3 B). To test the role of Rho GTPases (Rac, Cdc42, and Rho), we inhibited them all by treating cells with ﻿*Clostridium difficile* toxin B (Just et al., 1995) and found that the number of the WAVE complex rings formed after F-actin inhibition decreased significantly (Fig. 3 C). Furthermore, Pak-PBD (p21 activated kinase 1 protein–p21 binding domain), a biomarker for active Rac, broadly spatially overlaps with the WAVE complex’s discrete localization (Fig. 3 D), potentially representing a permissive zone for WAVE complex recruitment (Weiner et al., 2007). These data suggest that WAVE collaborates with upstream activators such as Rac in forming rings. We also explored whether the WAVE complex rings are templated by other ring-forming proteins such as clathrin and septins. Neither clathrin rings nor septin rings significantly colocalized with the WAVE complex rings (Fig. 3, E and F). Based on its lack of colocalization with clathrin, our data suggest that the WAVE complex is unlikely to participate in clathrin-mediated endocytosis, but we cannot exclude the possibility that it plays a role in clathrin-independent endocytosis. Strikingly, septins and the WAVE complex have mutually exclusive localization at the cell periphery (Fig. 3 F), potentially representing complementary curvature preferences.

### WAVE complex rings associate with saddle-shaped membrane geometry

**Figure 4.**
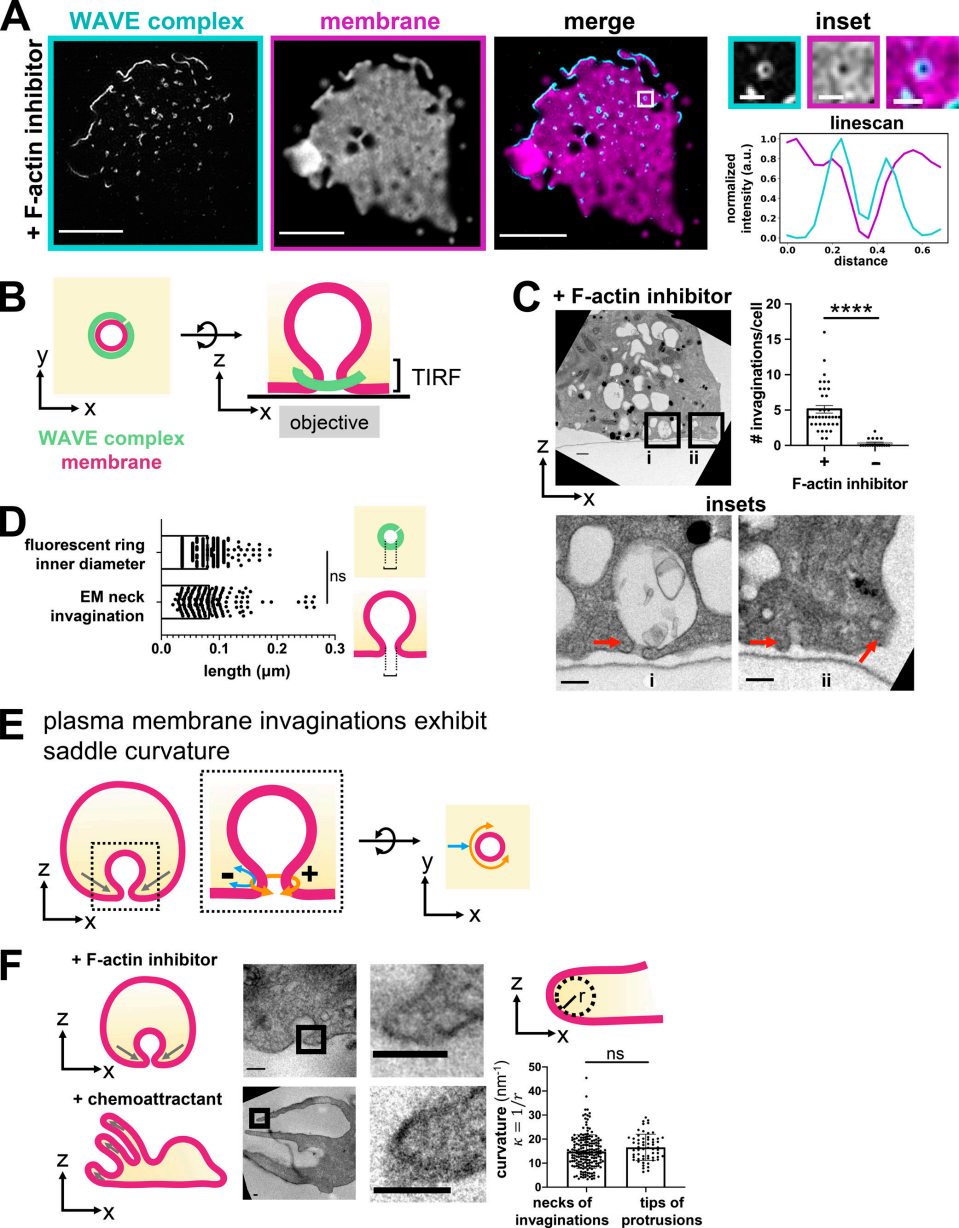
**WAVE complex rings associate with plasma membrane saddle points. (A)** TIRF-SIM imaging of the WAVE complex (Hem1-EGFP; cyan) reveals rings localized at the boundary where the plasma membrane (DiD-labeled; magenta) exits the TIRF-SIM plane. Right: Insets and a graph of a linescan across a ring. Scale bars: 5 µm and 500 nm (insets). See Video 3.** (B)** Schematic of the proposed organization of the WAVE complex ring structure (green) around the neck of a plasma membrane invagination (magenta) as seen from the top, x-y plane (left) or the side, x-z plane (right). **(C)** Transmission electron micrographs of a cross-section of dHL60s treated with latrunculin B (500 nM). Red arrows point to membrane invaginations. Insets (i and ii) shown below. Scale bars: 500 nm (top) and 200 nm (insets). Graph comparing the number of membrane invaginations per serial section of cells treated with or without F-actin inhibitor (500 nM latrunculin B). Graph shows mean ± SEM of the number of invaginations per cell; (+) F-actin inhibitor *n* = 37 cells, (−) F-actin inhibitor *n* = 18 cells; Mann–Whitney test with two-tailed; ****, P < 0.0001. **(D)** Graph comparing the length across the neck of invaginations by electron microscopy (EM) to the inner diameter of the fluorescent Hem1 rings of dHL60s treated with latrunculin B (500 nM). Schematic depicts where measurements were made. Graph shows mean ± SEM; EM of neck invaginations *n* = 190 from 38 cells, fluorescent ring inner diameter *n* = 222 from 4 cells; Mann–Whitney test with two-tailed; ns, not significant; P = ∼0.39 > 0.05. This finding supports the possibility that the WAVE complex enriches around the necks of membrane invaginations. **(E)** Schematic of a plasma membrane invagination’s saddle curvature. The necks of invaginations display saddle geometry of positive curvature in one axis (orange; the curve around the invagination neck) and negative curvature in the other axis (blue; the curve perpendicular to the invagination neck). **(F)** Curvature comparison of the necks of invaginations and tips of protrusions. Electron micrographs of dHL60s treated with F-actin inhibitor (top; 500 nM latrunculin B) and dHL60s treated with chemoattractant (bottom; 100 nM fMLP) show that the curvature at the necks of invaginations and the tips of protrusions are similar. Graph shows mean ± SEM; invaginations *n* = 190 from 28 cells, protrusions *n* = 59 from 19 cells; unpaired *t* test, two-tailed; ns, not significant; P = 0.074 > 0.05. Curvature, *κ*, defined as 1/r where r is the radius. Scale bar: 100 nm.

Next, we wondered how these WAVE complex rings are organized relative to the morphology of the plasma membrane. During cell migration, the WAVE complex is closely associated with the propagating edge of lamellipodial protrusions (Fig. 1 C). Dual imaging of the WAVE complex and the plasma membrane in the absence of F-actin revealed that the WAVE complex rings localized to the boundary where coverslip-opposed membrane leaves the TIRF field (Fig. 4 A and Video 3). This membrane distribution suggests an association of the WAVE complex to the necks of membrane invaginations (Fig. 4 B), and electron microscopy experiments are consistent with this plasma membrane geometry (Fig. 4, C and D; Galkina et al., 2018). Although we do not know the molecular composition of these invaginations, the WAVE complex’s propensity to enrich around the necks of plasma membrane invaginations may give insight into its membrane geometry preferences. The necks of membrane invaginations exhibit saddle curvature, principal curvatures that are positive in one axis (the curve around the invagination neck) and negative in the other axis (the curve perpendicular to the invagination neck; Fig. 4 E). Intriguingly, the negative curvature at the neck of an invagination is comparable to that at the tip of a protrusion (Fig. 4 F). In addition to upstream activators, saddle membrane geometry could potentially be another factor to proper WAVE complex localization.

**Video 3.** **TIRF-SIM imaging of WAVE complex rings at the necks of plasma membrane invaginations.** dHL60 cell expressing Hem1-EGFP (cyan) and stained with membrane dye (DiD; magenta) continually exposed to latrunculin B (500 nM). TIRF-SIM imaging at one frame every 10 s. This video corresponds to Fig. 4 A.

### The WAVE complex enriches to saddle-shaped transendothelial cell macroaperture (TEM) tunnels

**Figure 5.**
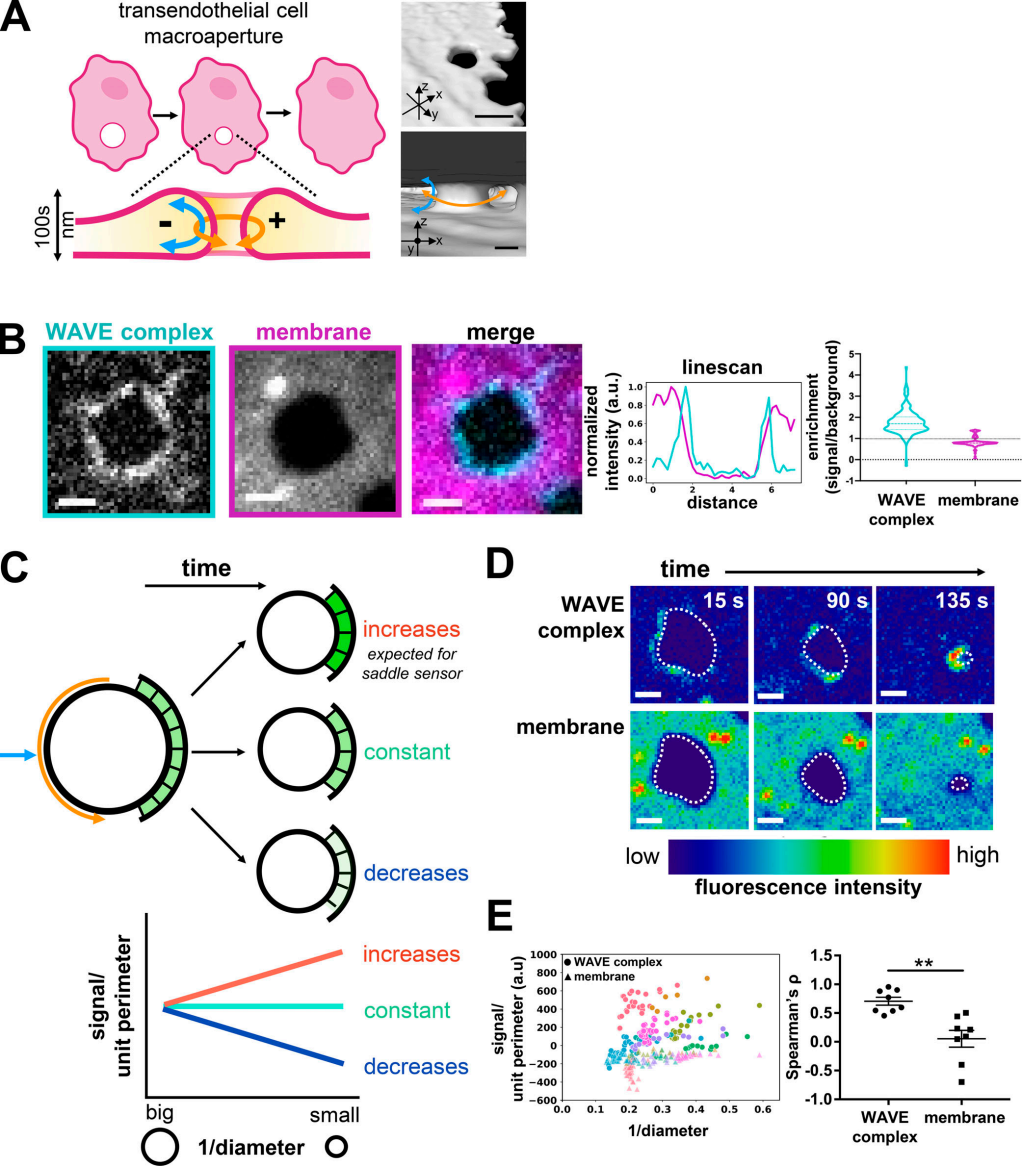
**WAVE complex enriches to TEM tunnel saddle points. (A)** Schematic of a TEM closure and saddle geometry. Right: ChimeraX renderings of spinning-disk confocal imaging of a HUVEC cell that was treated with Y27632 (50 µM) to induce TEMs, labeled with a membrane dye (CellMask DeepRed), and then fixed with paraformaldehyde. Top: Tilted view. Bottom: View from inside the cell facing a TEM and its saddle geometry. Scale bars: 5 µm (top) and 2 µm (bottom). **(B)** The WAVE complex localizes to TEMs. A HUVEC cell expressing EGFP-tagged Nap1, a WAVE complex subunit (cyan), and stained with membrane dye CellMask DeepRed (magenta) was treated with Y27632 (50 µM). Spinning-disk confocal imaging, scale bar: 2 µm. Left graph: Linescan across the TEM; WAVE complex (cyan) and membrane dye (magenta). Right graph: Violin plot of enrichment, which was measured as the ratio between the signal intensity per unit area at the TEMs compared with the background intensity per unit area. A value of 1 indicates no enrichment at the TEM (dotted line), and a value >1 indicates enrichment. Each time point throughout TEM closure was considered as a single data point; WAVE complex and membrane both had *n* = 164 from eight TEMs from at least three independent experiments per condition. **(C)** Schematic of different potential molecular behaviors during TEM closure. As TEMs close, the local concentration (signal per unit length) could increase, remain constant, or decrease. A saddle geometry sensor would increase its local concentration until the TEM closure reaches the sensor’s preferred positive radius of curvature. **(D)** Time-lapse images of the WAVE complex (top) and the membrane (bottom) as a TEM closes. Images in each set have the same intensity scale in a lookup table that eases the visualization of signal enrichment. The dotted line outlines the TEM membrane mask. Spinning-disk confocal imaging; time in seconds; scale bar: 2 µm. See Video 4.** (E)** WAVE complex enriches to closing TEMs. Left: Graph of the WAVE complex (circles) and membrane (triangles) signal per unit perimeter as a TEM closes. Each color represents a single TEM over time. The WAVE complex shows higher enrichment at smaller (more positively curved) TEMs, suggesting a preference for membrane saddles with high positive curvature. Right: Graph of mean ± SEM of the Spearman’s ρ correlation coefficient of each TEM shown on the left graph; the WAVE complex and membrane each had *n* = 8 TEMs from at least three independent experiments; unpaired *t* test, two-tailed; **, P = 0.0012 < 0.0021.

To further explore the possible role of saddle curvature recognition in the emergent control of cell shape, we took advantage of TEM tunnel physiology. As leukocytes undergo diapedesis out of the blood vessel, they can either migrate in between endothelial cells or generate a TEM, a transcellular hole, to migrate through endothelial cells (Carman and Springer, 2008). To heal the transcellular hole and prevent pathogen dissemination, the affected endothelial cell seals the macroaperture (Boyer et al., 2006; Lemichez et al., 2010; Maddugoda et al., 2011). The sealing process requires the recognition of the macroaperture, which exhibits saddle curvature, and subsequent closure of the hole (Fig. 5 A; Maddugoda et al., 2011). Closure is an Arp2/3 complex–mediated process, but the relevant Arp2/3 complex activator has not been identified (Maddugoda et al., 2011; Ng et al., 2017).

To study TEMs, we imaged the WAVE complex (via fluorescently tagged Nap1, a homologue of Hem1) in human umbilical vein endothelial cells (HUVECs; Carman and Springer, 2008; Maddugoda et al., 2011; Boyer et al., 2006; Ng et al., 2017) and found that it enriched to closing TEMs (Fig. 5 B and Video 4). This is another example of the WAVE complex’s association to saddle membrane geometry. An advantage of the TEM saddle system is that it maintains a fixed lamellipodial-like negative curvature in the z-plane while it scans a range of positive curvatures throughout its closure (Maddugoda et al., 2011; Stefani et al., 2017). Therefore, we can use this range of hole sizes to evaluate the positive curvature preference for the WAVE complex (Fig. 5 C). A saddle geometry sensor presented with fixed negative curvature in one axis and a range of positive curvatures in the other axis would increase its local concentration until the TEM closure reaches the sensor’s preferential positive curvature. From analyzing the WAVE complex signal throughout TEM closure, its local concentration per unit TEM perimeter increases at a rate significantly different from that of the membrane (Fig. 5, D and E). The WAVE complex’s progressive enrichment to smaller and smaller TEMs suggests that it can tolerate a range of positive curvatures but prefers smaller positive curvatures; this is consistent with our initial finding that the WAVE complex spontaneously forms sub-diffraction rings upon actin depolymerization (Fig. 2).

**Video 4.** **WAVE complex and IRSp53 at TEMs.** HUVECs expressing EGFP-tagged Nap1 (left), IRSp53 full-length (middle), or IRSp53’s I-BAR domain (right) were treated with Y27632 (50 µM). Imaged with spinning-disk confocal at one frame every 5 s. Scale bar: 10 µm. This video corresponds to Fig. 5 D and Fig. S4 D.

**Figure S3.**
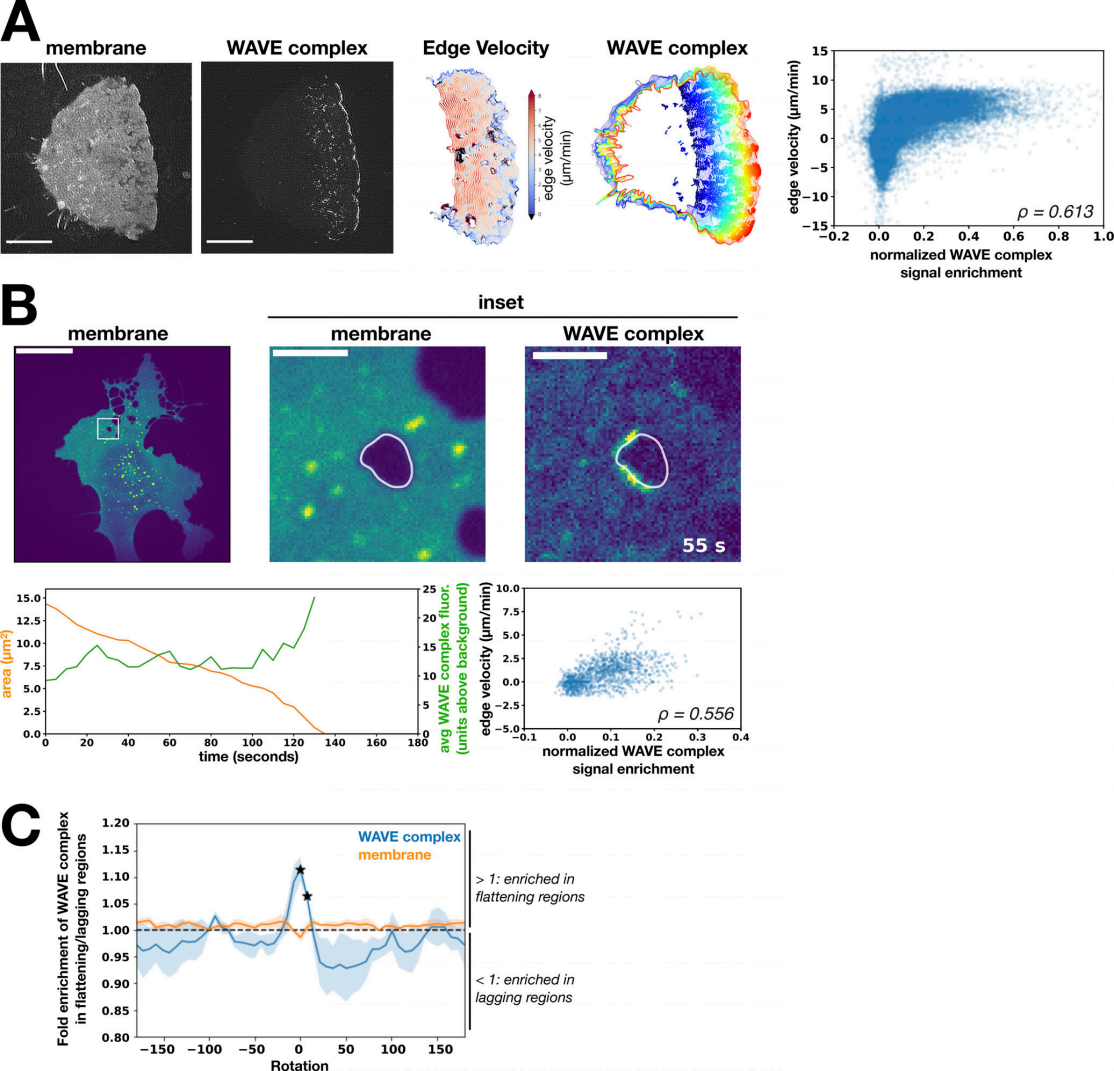
**The WAVE complex enriches at flattening membrane regions. (A)** WAVE complex signal enrichment versus membrane edge velocity in a migrating dHL60 cell. From left to right: Image of the membrane (CellMask DeepRed) at a single time point, image of the WAVE complex (Hem1-EGFP) at a single time point, successive cell outlines of the leading edge over time, WAVE complex localization (solid colors) over time, and graph of normalized WAVE complex signal enrichment versus membrane edge velocity of the entire membrane edge across all time points of the displayed cell (Spearman’s correlation ρ = 0.613). TIRF-SIM images; scale bar: 5 µm. **(B)** WAVE complex signal enrichment versus membrane edge velocity in a closing TEM. Images (top row): HUVEC cell expressing WAVE complex-EGFP (Nap1-EGFP) treated with Rho-associated protein kinase inhibitor Y27632 (50 µM) to generate TEMs. Left: membrane (CellMask DeepRed). Middle: Inset of membrane channel. Right: Inset of WAVE complex signal. Spinning-disk confocal imaging; scale bars: 40 µm and 5 µm (insets). Left graph: Area of TEM (orange) and the average WAVE complex fluorescence signal (units above background; green) over time (seconds). Right graph: Normalized WAVE complex signal enrichment versus membrane edge velocity at all time points across closure of the displayed transendothelial macroaperture (Spearman’s correlation ρ = 0.556). **(C)** The WAVE complex fold enrichment at flattening over lagging regions at different image rotations. “Flattening” is defined as the membrane becoming less positively curved (toward flat or negatively curved), “lagging” is defined as the membrane becoming more positively curved, and “rotation” refers to the degree to which the WAVE complex signal is rotated around the TEM. At each rotation, the fold enrichment of WAVE complex (blue) or membrane (orange) signal in flattening over lagging regions is calculated. The WAVE complex peak at zero rotation indicates that the highest enrichment was due to the colocalization of the curvature and fluorescent features of interest in the unrotated configuration, indicating a true correlation. The average ± SD of the enrichment scores for *n* = 3 TEMs were compared with the null value of 1 (no preference between flattening and lagging) using a two-tailed, nonpaired *t* test at each rotation value. A particular rotation was marked significant with a star if P < 0.05. avg, average; fluor., fluorescence.

Furthermore, we found that the WAVE complex enrichment correlates with membrane velocity; regions of membrane with more WAVE complex correlate with higher velocity for both advancing neutrophil leading edges and closing transendothelial macroapertures (Fig. S3, A and B). As membranes protrude in these cases, some regions may transiently fall behind or “lag.” To maintain a coherent advancing front, the lagging portion, which exhibits positive curvature, must become less positively curved, i.e., flatten/straighten, and accelerate to “catch up.” The WAVE complex preferentially enriches to transendothelial macroaperture membrane regions that are flattening compared with those that are becoming more curved (Fig. S3 C). These data suggest that the WAVE complex prefers nanoscale saddle geometry in a range of cellular and physiological contexts and support a self-straightening role for WAVE-mediated protrusions.

### The WAVE complex recruits IRSp53 to lamellipodia and saddle curvature

**Figure 6.**
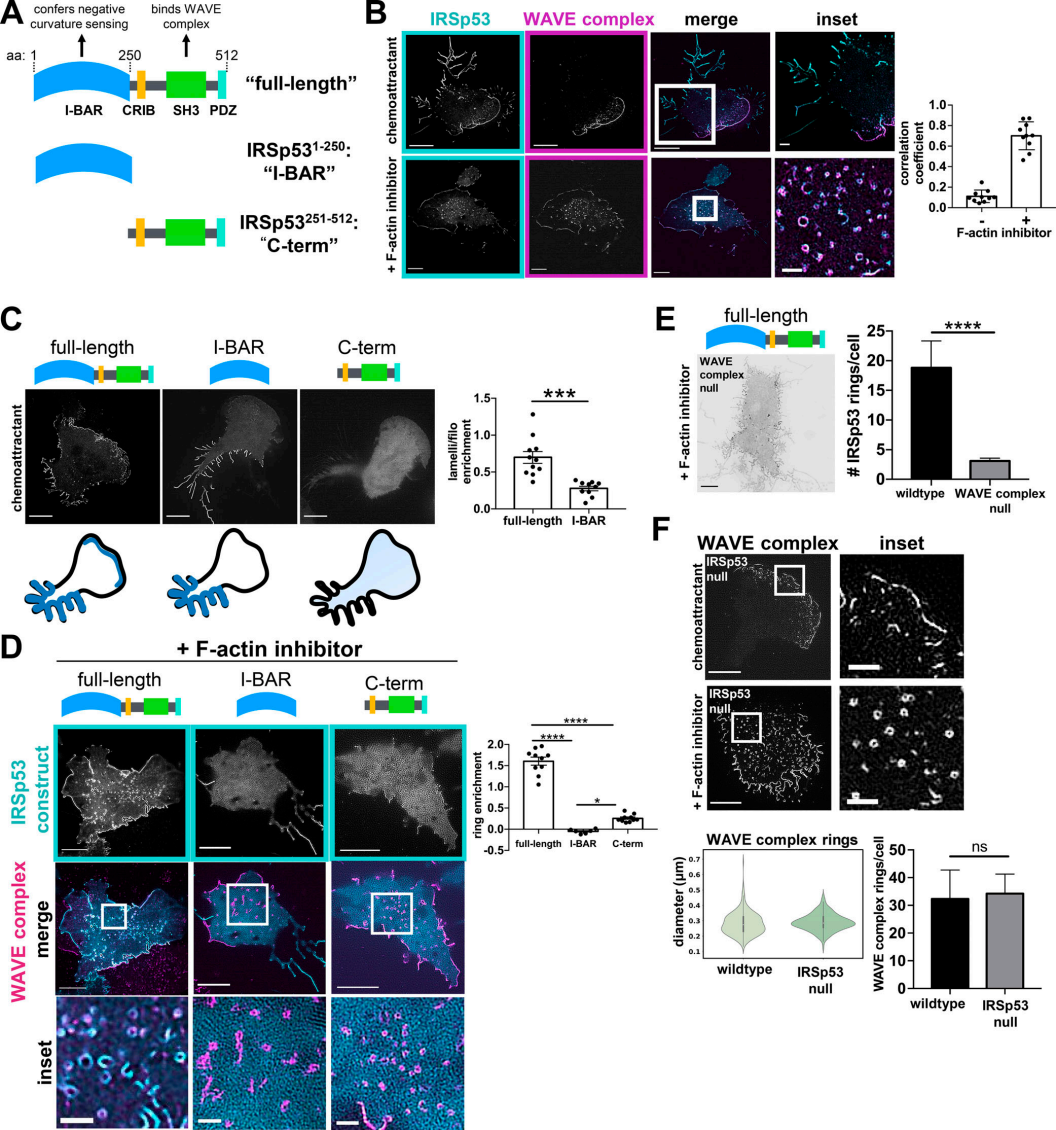
**WAVE complex recruits IRSp53 to lamellipodia and regions of saddle curvature. (A)** Domain structure of IRSp53 structure/function constructs. The I-BAR domain confers negative membrane curvature sensing and the SH3 domain enables binding to the WAVE complex. **(B)** IRSp53 and the WAVE complex colocalize at lamellipodia (top). Following actin depolymerization, they colocalize at rings (bottom). IRSp53 also localizes to filopodia-like structures, whereas the WAVE complex is excluded from those regions (top). dHL60 cells expressing both IRSp53-EGFP (cyan) and Hem1-mCherry (magenta) were treated with chemoattractant (10 nM fMLP, top) or F-actin inhibitor (500 nM latrunculin B, bottom). TIRF-SIM imaging; scale bars: 5 µm and 1 µm (insets). See Video 5. Graph measuring colocalization between IRSp53 and Hem1 in conditions with or without F-actin inhibition. Barplot shows mean ± SD of Manders M1 correlation coefficient (fraction of IRSp53 in compartments containing Hem1); (+) F-actin inhibition *n* = 10 cells, (−) F-actin inhibition *n* = 10 cells; cells pooled from at least three independent experiments of TIRF-SIM imaging. **(C)** The IRSp53 structure/function constructs have different localization patterns in chemoattractant-stimulated dHL60s. Full-length IRSp53 enriches to lamellipodia and filopodia-like structures; I-BAR enriches to filopodia-like structures but not to lamellipodia; and C-term is cytosolic. Summary of the IRSp53 construct localization below. Cells with comparable expression levels were imaged (Fig. S4 C). TIRF-SIM imaging; scale bar: 5 µm. Right: Graph shows mean ± SEM of the IRSp53 enrichment ratio (IRSp53 signal at lamellipodia per unit area versus IRSp53 signal at filopodia per unit area); full-length *n* = 11 cells; I-BAR *n* = 10 cells; cells pooled from at least three independent experiments per condition; unpaired *t* test, two-tailed; ***, P = 0.0002 < 0.001. **(D)** F-actin inhibited dHL60s expressing IRSp53 constructs and the WAVE complex. Middle row shows IRSp53-EGFP construct (cyan) overlay with Hem1-mCherry (magenta) rings. The I-BAR and C-term constructs fail to enrich robustly as rings. TIRF-SIM imaging; scale bars: 5 µm and 1 µm (insets). Graph comparing the signal enrichment of IRSp53 constructs’ signal per unit area of rings (defined by Hem1-mCherry) over the background per unit area. Graph displays the mean ± SEM where all rings within a cell were aggregated; full-length *n* = 10 cells from three independent experiments, I-BAR *n* = 6 cells from two independent experiments, C-term *n* = 10 cells from two independent experiments; one-way ANOVA with two-tailed; P < 0.0001 with Tukey’s multiple comparisons follow up tests, ****, P < 0.0001; *, P = 0.01 < 0.05. **(E)** Full-length IRSp53 fails to enrich as rings in the absence of the WAVE complex. Inverted display of IRSp53-EGFP expressed in a WAVE complex-null dHL60 cell treated with latrunculin B (500 nM). TIRF-SIM imaging; scale bar: 5 µm. Graph comparing the mean ± SEM of the number of IRSp53 rings in wild type and WAVE complex-null cells; wild type *n* = 37 cells, WAVE complex-null *n* = 66 cells; cells pooled from the same three independent experiments; unpaired *t* test, two-tailed; ****, P < 0.0001. **(F)** The WAVE complex in IRSp53-null dHL60 cells. The WAVE complex localizes to lamellipodia in chemoattractant-stimulated cells (10 nM fMLP; top) and as rings in F-actin–inhibited cells (500 nM latrunculin B; bottom). TIRF-SIM imaging; scale bars: 5 µm and 1 µm (inset). Left graph: Violin plot of the WAVE complex rings in wild type and IRSp53-null cells; wild type: *n* = 293 rings from 13 cells, IRSp53-null: *n* = 470 rings from 10 cells; cells pooled from at least three independent experiments per condition. Diameters were measured in the same fashion shown in Fig. 2 D. Right graph: Graph comparing the mean ± SEM number of the WAVE complex rings in wild type and IRSp53-null cells; wild type, *n* = 10 cells, IRSp53-null *n* = 16 cells; cells pooled from the same three independent experiments; unpaired *t* test, two-tailed; ns, not significant; P = 0.88 > 0.05.

Next, we sought to understand how the WAVE complex localizes to saddle geometries. Though the WAVE complex has membrane-binding motifs (Oikawa et al., 2004), it lacks any well-characterized curvature-sensing motifs. However, the WAVE complex does directly interact with IRSp53 (human orthologue is brain-specific angiogenesis inhibitor 1–-associated protein 2 [BAIAP2]), a member of the I-BAR domain family of curvature-sensitive proteins that associates with lamellipodia and filopodia (Fig. 6 A; Suetsugu et al., 2006; Abou-Kheir et al., 2008; Miki et al., 2000; Scita et al., 2008; Frost et al., 2009; Zhao et al., 2011; Mattila et al., 2007).

**Figure S4.**
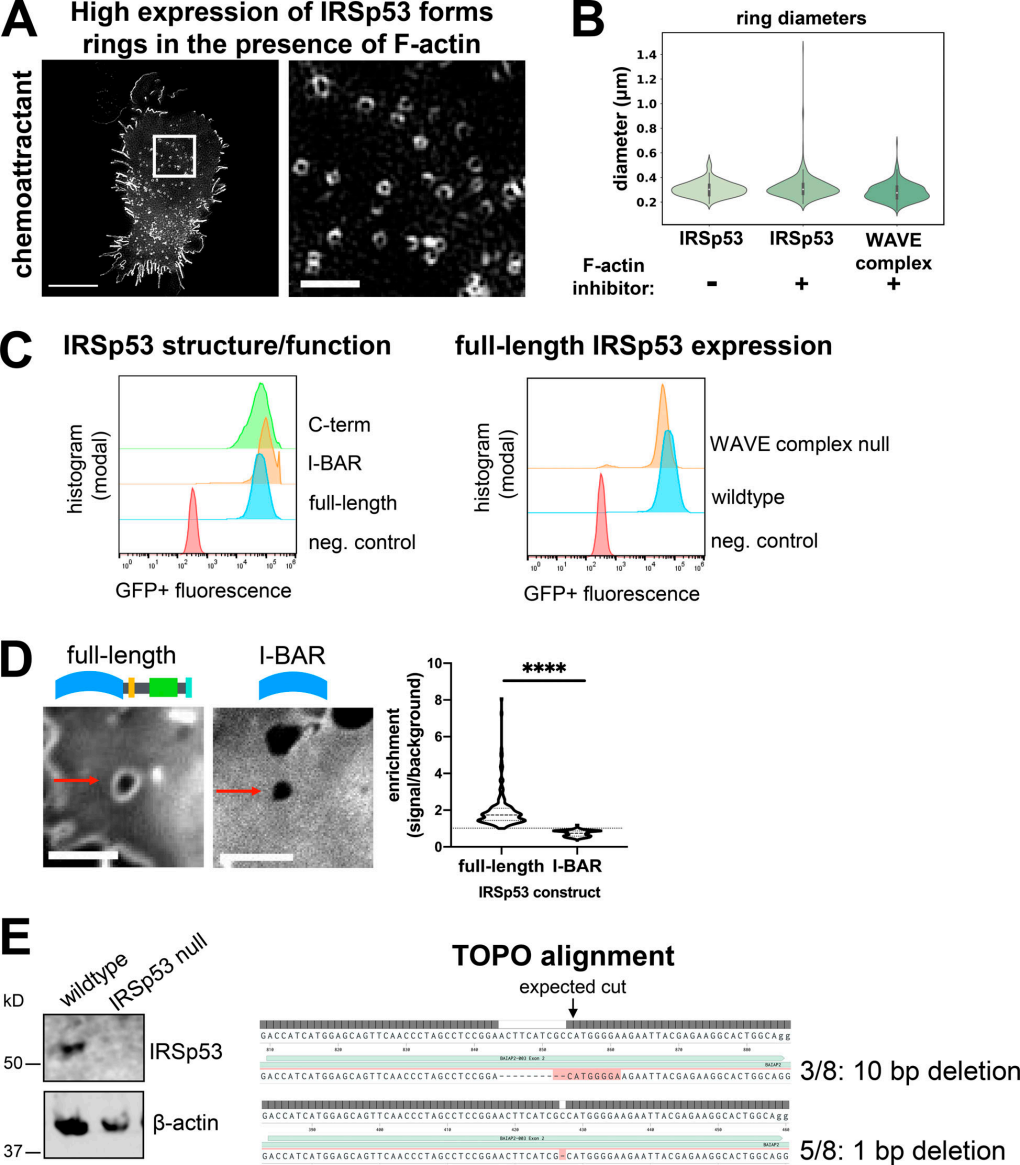
**Supplemental IRSp53 data. (A)** dHL60 cell with high expression of IRSp53-EGFP stimulated with chemoattractant (10 nM fMLP) shows that high levels of IRSp53 induce ring structures even in the presence of an intact actin cytoskeleton. TIRF-SIM imaging; scale bars: 5 µm (left) and 1 µm (inset). See Video 6.** (B)** Graph comparing the diameters of the WAVE complex rings with F-actin inhibition to IRSp53 rings with (+) or without (−) F-actin inhibition (500 nM latrunculin B). Diameters measured in the same fashion shown in Fig. 2 D. Violin plot of rings; IRSp53 without (−) F-actin inhibition *n* = 157 from 10 cells, IRSp53 with (+) F-actin inhibition *n* = 483 from 10 cells, the WAVE complex (+) F-actin inhibition *n* = 202 from 13 cells; cells pooled from at least three independent experiments per condition; Kruskal–Wallis test; nonsignificant P = 0.56 > 0.05. **(C)** IRSp53-EGFP structure/function expression levels are comparable across cell lines. Left: IRSp53 structure/function constructs in wild type cells. Right: Full-length IRSp53 in wild type and WAVE complex-null HL60s. Flow cytometry data graphed with FlowJo. **(D)** Images of HUVECs expressing full-length IRSp53 (left) or I-BAR–only domain (right) at Y27632-induced TEMs. Spinning-disk confocal imaging; red arrows point to a TEM; scale bar: 5 µm. See Video 4. Graph: Violin plot of the enrichment of full-length IRSp53 and I-BAR domain at TEMs; measurements made in the same fashion as Fig. 5 B. Each time point across TEM closure was considered as a single data point: full-length *n* = 186 and I-BAR *n* = 291, both from four TEMs from at least three independent experiments per condition; Mann–Whitney test of significance; two-tailed; ****, P < 0.0001. **(E)** Characterization of CRISPR/Cas9-mediated IRSp53 knockout in HL60s. Left: Immunoblot of IRSp53 and β-actin (loading control) in wild type and IRSp53-null clonal HL60 line. Right: TOPO alignment of the gene-edited region of exon 2 reveals indels of the IRSp53-null clonal HL60 line. neg., negative.

To compare their localization patterns, we imaged IRSp53 and the WAVE complex in dHL60 cells. Full-length IRSp53 localized to multiple areas of negative curvature: lamellipodia (Suetsugu et al., 2006; Abou-Kheir et al., 2008; Miki et al., 2000; Nakagawa et al., 2003; Robens et al., 2010), filopodia-like tubules (Nakagawa et al., 2003; Robens et al., 2010; Krugmann et al., 2001; Sudhaharan et al., 2019), and rings (Fig. 6 B and Fig. S4 A). IRSp53 and the WAVE complex only colocalized at protruding lamellipodia during cell migration; WAVE complex was absent at filopodial-like tubules (Fig. 6 B, top; and Video 5). When cells were treated with a F-actin inhibitor, both the WAVE complex and IRSp53 colocalized to rings (Fig. 6 B, bottom), consistent with both proteins showing saddle curvature enrichment. Strikingly, high expression of IRSp53 induces ring structures even without F-actin inhibition (Fig. S4 A and Video 6), suggesting a potential functional interaction of these two proteins at sites of saddle curvature. IRSp53 rings (± F-actin inhibitor) had comparable diameters to the WAVE complex rings (+ F-actin inhibitor; Fig. S4 B). This characteristic size suggests a preferred geometry for IRSp53 and the WAVE complex’s membrane recruitment in cells. While IRSp53 localized to multiple sites displaying negative curvature, the WAVE complex was specifically enriched to lamellipodia and regions of saddle curvature.

**Video 5.** **TIRF-SIM imaging of IRSp53 and WAVE complex colocalization at a protruding lamellipodium.** dHL60 cell expressing IRSp53-EGFP (cyan, left) and Hem1-mCherry (magenta, middle) continually exposed to chemoattractant (10 nM fMLP) imaged with TIRF-SIM at one frame every 5 s. Merged channels (right) show colocalization at lamellipodium edge. Scale bar: 5 µm. This video corresponds to Fig. 6 B, top.

**Video 6.** **TIRF-SIM imaging of the induction of IRSp53 ring structures in the presence of F-actin.** dHL60 cell with high expression of IRSp53-EGFP continually exposed to chemoattractant (10 nM fMLP) imaged with TIRF-SIM at one frame every 5 s. Scale bar: 5 µm. This video corresponds to Fig. S4 A.

Since the WAVE complex and IRSp53 colocalize in a subset of cell structures, we wondered whether IRSp53’s localization pattern may be partially dependent on its interactions with the WAVE complex. IRSp53 consists of several functional domains that could contribute to its localization, in particular an I-BAR domain that senses negative curvature and an Src homology 3 (SH3) domain that interacts with the WAVE complex (Suetsugu et al., 2006; Abou-Kheir et al., 2008; Miki et al., 2000). To investigate how these domains contribute to the overall pattern of IRSp53 enrichment, we generated two structure/function constructs tagged with EGFP at the C terminus: an “I-BAR”–only domain that consisted of IRSp53’s first 250 amino acids, and a “C-term”–only construct that lacks the I-BAR domain but contains the region that interacts with the WAVE complex (Fig. 6 A and Fig. S4 C). In chemoattractant-stimulated cells, full-length IRSp53 enriched to both lamellipodia and filopodia-like structures, the I-BAR domain preferentially enriched to filopodia-like structures, and the C-term domain remained cytosolic (Fig. 6 C). In F-actin–inhibited cells, full-length IRSp53 formed rings, whereas the I-BAR and C-term constructs failed to enrich as rings (Fig. 6 D). These data suggest that IRSp53 requires both its I-BAR domain and its C-terminal portion, containing its SH3-WAVE complex interacting domain, to properly enrich to lamellipodia and saddle points.

Since IRSp53’s SH3 domain interacts with other actin regulators besides the WAVE complex (including Mena [Krugmann et al., 2001], Eps8 [Disanza et al., 2006], and mDia1 [Goh et al., 2012]), we tested whether IRSp53’s localization patterns are dependent on the WAVE complex by imaging full-length IRSp53 in WAVE complex–null cells. The WAVE complex was depleted via CRISPR/Cas9-mediated knockout of Hem1, which resulted in the depletion of the other subunits (Leithner et al., 2016; Graziano et al., 2019; Litschko et al., 2017). Upon F-actin inhibition, full-length IRSp53 failed to robustly form rings in the WAVE complex–null cells (Fig. 6 E). These data suggest that while IRSp53’s I-BAR domain is sufficient to enrich to filopodia-like structures, IRSp53 requires its I-BAR domain and its interactions with the WAVE complex to localize to lamellipodia and saddle geometries. Our data highlight how proteins can partner together to sense complex geometries.

To probe the generality of IRSp53 requiring both its I-BAR domain and its ability to bind to the WAVE complex to sense saddle curvature, we returned to the HUVEC TEM system. A previous study found that IRSp53’s isolated I-BAR domain fails to recognize TEMs (Maddugoda et al., 2011), and we confirmed these results in our hands (Fig. S4 D and Video 4). This is consistent with our observation that the isolated I-BAR construct failed to enrich saddle-shaped membrane invaginations in dHL60s (Fig. 6 D). Because full-length IRSp53 recognized saddle curvature in dHL60 cells (Fig. 6 D), we expected that full-length IRSp53 would also recognize TEMs, and our observations are consistent with this hypothesis (Fig. S4 D and Video 4). These data show that IRSp53 depends on its ability to interact with the WAVE complex to localize to saddle geometries in a range of cellular contexts.

### Which WAVE effectors are essential to instruct lamellipodial assembly?

The WAVE complex has several effectors and associated proteins it could use to instruct lamellipodial assembly (Fig. 1 A). Here we investigate the role of two such effectors (IRSp53 and the Arp2/3 complex) in lamellipodial formation. IRSp53 requires the WAVE complex to localize to lamellipodia and saddle geometries (Fig. 6, C–E). To test whether the converse is true, we imaged the WAVE complex in dHL60 cells following CRISPR/Cas9-mediated knockout of IRSp53 (Fig. S4 E). The absence of IRSp53 did not affect the ability of the WAVE complex to localize to lamellipodia or alter the number or size of WAVE complex rings following actin depolymerization (Fig. 6 F). These data suggest that the WAVE complex does not depend on its partnering interactions with IRSp53 to localize to lamellipodia and saddle curvature. We do not know if other I-BAR proteins are involved in recruiting the WAVE complex to sites of saddle curvature or if other mechanisms are at play.

**Figure 7.**
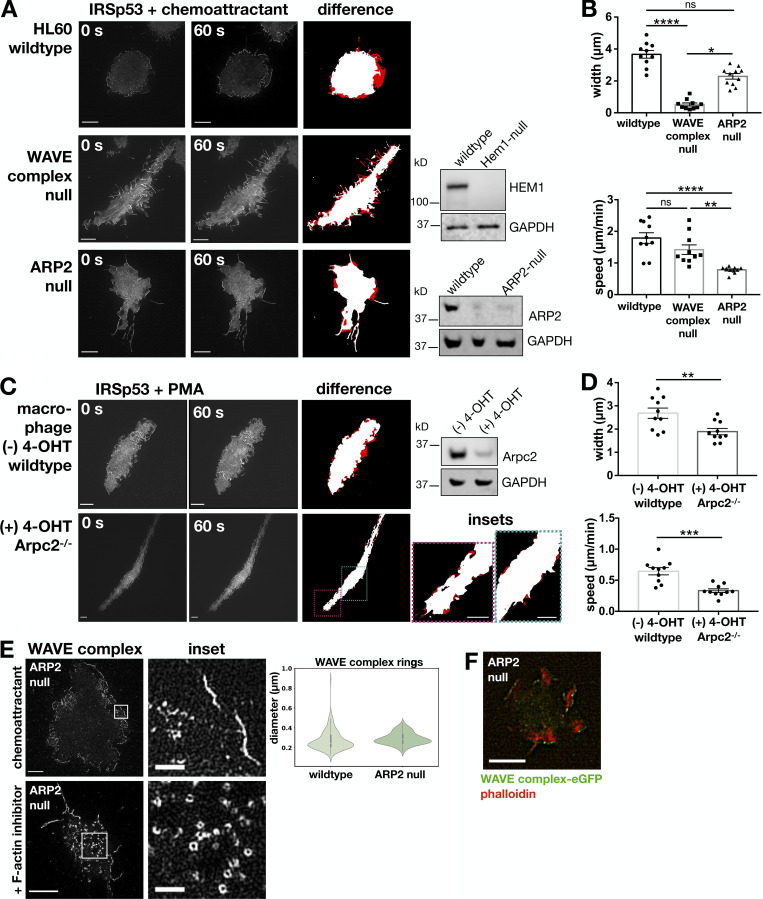
**The WAVE complex, but not the Arp2/3 complex, is required for the formation of sheet-like protrusions. (A)** Arp2/3 complex-disrupted dHL60 cells form sheet-like protrusions. IRSp53, a marker of both filopodial and lamellipodial protrusions, was imaged in chemoattractant-stimulated wild type (top row), WAVE complex-null (middle row), and ARP2-null (bottom row) cells. Middle column panel is 60 s after chemoattractant stimulation of the corresponding left column panel. Right column panel displays the difference (red) to highlight regions of protrusions. TIRF-SIM imaging; scale bar: 5 µm. See Video 7. Western blots for HEM1 and ARP2 with GAPDH as a loading control are shown for wild type and CRISPR/Cas9-edited cells. **(B)** Graphs comparing the protrusions across cells. Top graph: Mean ± SEM of the average protrusion widths (microns) after 1 min of chemoattractant stimulation per cell; wild type *n* = 10 cells, WAVE complex-null *n* = 10 cells, and ARP2-null *n* = 11 cells; cells pooled from at least three independent experiments per condition; Kruskal–Wallis test; P < 0.0001 with Dunn’s multiple comparisons follow up tests; ****, P < 0.0001; *, P = 0.0141; ns, not significant; P = 0.0717 > 0.05. Bottom graph: Mean ± SEM of the average protrusion speed (micron per minute) after chemoattractant stimulation per cell; wild type *n* = 10 cells, WAVE complex-null *n* = 10 cells, and ARP2-null *n* = 11 cells; cells pooled from at least three independent experiments per condition; Kruskal–Wallis test; P < 0.0001 with Dunn’s multiple comparisons follow-up tests; ****, P < 0.0001; **, P = 0.0029; ns, not significant; P > 0.99. **(C)** *Arpc2*-disrupted primary mouse bone marrow macrophages form sheet-like protrusions. IRSp53-EGFP was imaged in PMA-stimulated (100 nM) wild type ([−] 4-OHT) and *Arpc2*^−/−^ ([+] 4-OHT) cells. Panels displayed similarly to A. TIRF-SIM imaging; scale bar: 5 µm. See Video 8. Western blot for Arpc*2* and GAPDH as a loading control is shown. **(D)** Graphs comparing the protrusions across macrophages. Top graph: Mean ± SEM of the average protrusion widths (microns) after 1 min of PMA stimulation per cell; wild type ([−] 4-OHT) *n* = 10 cells and *Arpc2*^−/−^ ([+] 4-OHT) *n* = 10 cells; cells pooled from two experiments per condition; Mann–Whitney test; **, P = 0.0052. Bottom graph: Mean ± SEM of the average protrusion speed (micron per minute) after PMA stimulation per cell; wild type ([−] 4-OHT) *n* = 10 cells and *Arpc2*^−/−^ ([+] 4-OHT) *n* = 10 cells; cells pooled from two experiments per condition; Mann–Whitney test; ***, P = 0.0002. **(E)** The WAVE complex in ARP2-null dHL60 cells. The WAVE complex (Hem1-EGFP) localizes to sheet-like protrusions in chemoattractant-stimulated ARP2-null cells (10 nM fMLP; top) and as rings in F-actin–inhibited ARP2-null cells (500 nM latrunculin A; bottom). TIRF-SIM imaging; scale bars: 5 µm and 1 µm (insets). See Video 9. Violin plot of the WAVE complex ring diameters in wild type and ARP2-null cells; wild type *n* = 210 from 10 cells, ARP2-null *n* = 179 from 10 cells; cells pooled from at least three independent experiments per condition. Diameters measured in the same fashion shown in Fig. 2 D. **(F)** F-actin behind the WAVE complex in ARP2-null sheet-like protrusions. ARP2-null dHL60 cell expressing Hem1-EGFP (green) treated with chemoattractant (100 nM fMLP) and stained with phalloidin (red). TIRF imaging; scale bar: 5 µm.

Though it is known that the WAVE complex is required to form sheet-like protrusions (Weiner et al., 2006; Leithner et al., 2016; Graziano et al., 2019), it is unclear what aspect of the WAVE complex activity is essential. One of the most well-established links from the WAVE complex to the actin cytoskeleton is its activation of the Arp2/3 complex (Machesky and Insall, 1998; Pollard and Borisy, 2003; Weaver et al., 2003); though the WAVE complex has other Arp2/3 complex–independent links to actin polymerization (Bieling et al., 2018). To investigate the necessity of this link for sheet-like protrusions, we compared the TIRF-SIM localization of IRSp53, a marker of both filopodial and lamellipodial protrusions, in chemoattractant-stimulated WAVE complex–null and ARP2-null dHL60 cells (Fig. 7, A and B; and Video 7; Graziano et al., 2019) as well as in *Arpc2*^−/−^ primary mouse bone marrow macrophages (Fig. 7, C and D; and Video 8; Rotty et al., 2017). Depletion of the WAVE complex resulted in the loss of broad, protrusive lamellipodia structures (Fig. 7 A, middle). In contrast, cells with Arp2/3 complex disrupted still form sheet-like structures (Fig. 7 A, bottom) in which the WAVE complex localizes to the protruding edges (Fig. 7 E and Video 9) ahead of F-actin–rich accumulations (Fig. 7 F). The ARP2-null sheet-like protrusions extend more slowly compared with wild type protrusions (Fig. 7 B), suggesting that the Arp2/3 complex contributes to the rate of lamellipodial advancement. To test the generality of the ability of cells to build sheet-like protrusions in the absence of the Arp2/3 complex, we imaged IRSp53 in primary macrophages that have an *Arpc2*/p34 subunit conditional allele (Fig. 7 C and Video 8; Rotty et al., 2015, 2017). When the macrophages were treated with 4-hydroxy-tamoxifen (4-OHT) to induce the CreER-dependent deletion of *Arpc2*, we observed elongated cell morphologies that also formed sheet-like protrusions that extend slowly compared with wild type cells (Fig. 7, C and D). The difference in morphology between WAVE complex–null and Arp2/3-disrupted cells suggests that the WAVE complex has roles in sheet-like protrusion formation beyond its activation of the Arp2/3 complex, possibly reflecting the actin polymerase role of the WAVE complex (Bieling et al., 2018).

**Video 7.** **Sheet-like protrusions in ARP2-disrupted dHL60 cells.** IRSp53-EGFP localization in chemoattractant stimulated (10 nM fMLP) wild type (left), WAVE complex-null (middle), and ARP2-null (right) dHL60 cells. IRSp53 is a marker of both filopodial and lamellipodial protrusions. TIRF-SIM imaging at one frame every 4 s for wild type cells and one frame every 5 s for WAVE complex and ARP2 nulls. Scale bar: 5 µm. This video corresponds to Fig. 7 A.

**Video 8.** **Sheet-like protrusions in *Arpc2*^−/−^ macrophage cells.** IRSp53-EGFP localization in stimulated (100 nM PMA; −) 4-OHT wild type (left) and (+) 4-OHT *Arpc2*^−/−^ (right) macrophage cells. TIRF-SIM imaging at one frame every 10 s. Scale bar: 10 µm. This video corresponds to Fig. 7 C.

**Video 9.** **The WAVE complex localization in ARP2-disrupted dHL60 cells.** The WAVE complex (Hem1-EGFP) localization in an ARP2-null dHL60 cell stimulated with chemoattractant (10 nM fMLP). TIRF-SIM imaging at one frame every 5 s. Scale bar: 5 µm. This video corresponds to Fig. 7 E, top.

**Figure S5.**
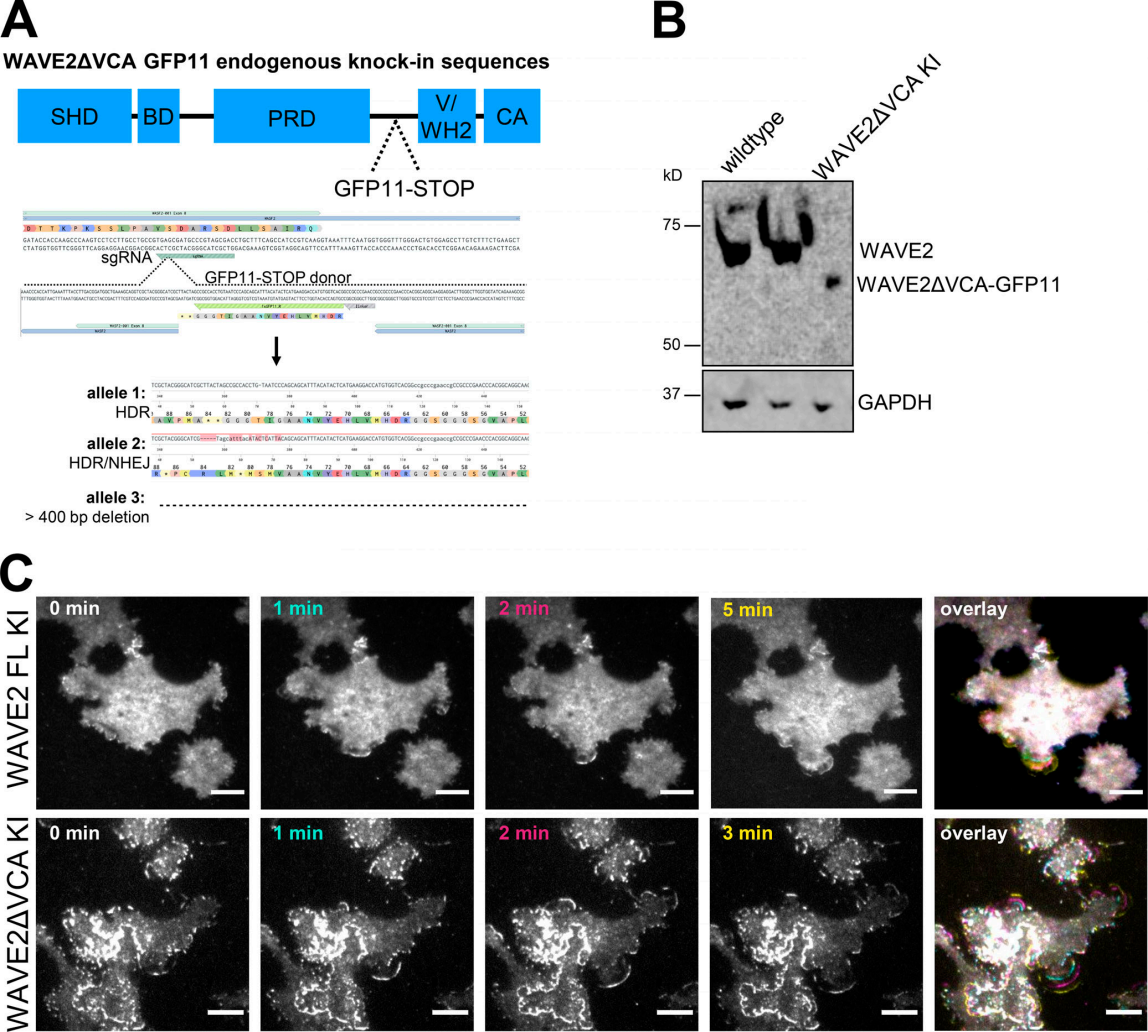
**WAVE2ΔVCA localizes to the leading edge of sheet-like protrusions. (A)** Generation of WAVE2ΔVCA cells with the split-GFP endogenous knock-in CRISPR/Cas9 editing system. Schematic of WAVE2 domains and editing (SHD, scar homology domain; BD, basic domain; PRD, proline-rich domain; V/WH2, WASP-homology 2 domain; CA, central acidic domain). In a GFP1-10–expressing cell line, CRISPR/Cas9 with a guide targeting WAVE2’s VCA region cuts and the cell repairs with a homology-directed repair (HDR) donor containing GFP11 and two stop codons. After generating clonal cell lines, the edited region was PCR-amplified and analyzed with MiSeq and CRISPresso2. The edited alleles are one HDR allele, one mixed HDR/NHEJ allele (which resulted in a stop codon), and one allele with a deletion >400 bp. **(B)** Western blot of WAVE2 and GAPDH in wild type and WAVE2ΔVCA GFP11 knock-in HEK-293T lines. **(C)** WAVE2ΔVCA localizes to the leading edge of sheet-like protrusions. Time lapse of endogenously split-GFP–edited full-length WAVE2 (FL KI, top row) and WAVE2ΔVCA (ΔVCA KI, bottom row) HEK-293T cells stimulated with 300 nM PMA. TIRF imaging; scale bar: 10 µm. See Video 10.

To further probe the WAVE complex’s Arp2/3 complex–independent roles, we generated an endogenous WAVE2ΔVCA split-GFP knock-in HEK-293T cell line (Fig. S5, A and B). We found that WAVE2ΔVCA localizes to the leading edge of sheet-like protrusions (Fig. S5 C and Video 10), thus highlighting the WAVE complex’s central role in organizing lamellipodia beyond its connection to the Arp2/3 complex.

**Video 10.** **WAVE2ΔVCA localizes to the leading edge of sheet-like protrusions.** WAVE2ΔVCA split-GFP endogenous knock-in localization in HEK-293T cells stimulated with PMA (300 nM). TIRF imaging at one frame every 30 s. Scale bar: 10 µm. This video corresponds to Fig. S5 C, bottom.

## Discussion

### The WAVE complex assembles into a linear, curved oligomer at sites of saddle membrane curvature

In this work, we investigated how the WAVE complex achieves the proper spatial organization for building lamellipodia. We found that the WAVE complex assembles into highly ordered nanoscale rings in the absence of actin polymer (Fig. 2). The WAVE complex rings are dependent on upstream regulators like Rac (Fig. 3) Furthermore, the rings associate with regions of saddle curvature at the plasma membrane (Fig. 4). The WAVE complex’s enrichment to membrane saddles is also apparent in closing TEMs (Fig. 5). IRSp53 requires its interactions with the WAVE complex to localize to lamellipodia, membrane invaginations, and TEMs, but the converse is not true (Fig. 6). Furthermore, sheet-like protrusions form in Arp2/3 complex–disrupted cells but not in WAVE complex–null cells (Fig. 7). This suggests that the WAVE complex’s link to lamellipodial assembly is not limited to the activation of the Arp2/3 complex, underscoring the central role of the WAVE complex in generating lamellipodia. The WAVE complex’s association with saddle-shaped membrane coupled with its regulation of actin polymerization likely forms the basis for emergent self-organizing behaviors like cell morphogenesis during migration and closure of transcellular holes.

How the WAVE complex achieves its oligomeric association into stereotyped 230-nm rings is unknown. There is no evidence that the purified WAVE complex oligomerizes on its own (Chen et al., 2010). Even in the presence of its known coactivators and with a complete motility mix (or even the presence of cytosol), there have been no WAVE complex rings, lines, propagating dynamics, or self-organizing lamellipodia observed (Koronakis et al., 2011; Lebensohn and Kirschner, 2009; Chen et al., 2010). It is likely that the WAVE complex requires its interacting partners to form higher-order oligomers, as is the case for N-WASP (Case et al., 2019; Banjade and Rosen, 2014). It is possible that the same proteins that activate the nucleation ability of the WAVE complex, such as phosphoinositides, Rac, and Arf (Koronakis et al., 2011; Lebensohn and Kirschner, 2009; Suetsugu et al., 2006; Chen et al., 2017), or post-translational modifications of the complex (Ura et al., 2012; Mendoza et al., 2011; Singh and Insall, 2021) may be needed to activate WAVE complex oligomerization. Consistent with this idea, we observe an increase in WAVE complex rings following chemoattractant stimulation of latrunculin-treated cells (Fig. 3 B), and Rho family GTPase inhibition inhibits WAVE complex ring formation (Fig. 3 C).

The WAVE complex’s oligomerization into rings may contribute to how it recognizes sites of plasma membrane saddle geometry. Given the stereotypical ring diameter of 230 nm (Fig. S2), the enrichment to membrane saddles with near-diffraction-limited curvature in the positive axis for closing TEMs (Fig. 5 E), and the overexpression of IRSp53 generating similarly sized rings in the presence of an intact actin cytoskeleton (Fig. S4, A and B), these data are consistent with a radius of positive curvature preference of 115 nm. What is the dimension of the WAVE complex rings in the negative curvature axis? Although we cannot visualize this directly in our TIRF-SIM images of F-actin–inhibited cells (negative curvature axis is in the z-plane, where TIRF provides no information), our electron microscopy imaging of membrane invaginations gives a height estimate of ∼130 nm, which is consistent with the negative curvature of the edge of lamellipodia in our work (Fig. 4 F) and others’ work (Abercrombie et al., 1970; Abraham et al., 1999; Urban et al., 2010), and in agreement with the at most ∼100-nm resolution of TIRF-SIM imaging of propagating WAVE complex in non–F-actin–inhibited cells. These data suggest a radius of negative curvature preference of 65 nm.

Ring formation has been observed for other proteins that sense plasma membrane curvature. For example, septins form rings in vivo following treatment with actin-destabilizing drugs, and purified septins form similarly sized rings in vitro (Kinoshita et al., 2002). The radius of curvature of septin rings reflects septins’ intrinsic curvature preference both in vitro and in vivo (Kinoshita et al., 2002; Bridges et al., 2016). Similar to septins, the WAVE complex also forms ring structures upon actin depolymerization, and these rings associate with curved membranes, although the WAVE complex rings fail to colocalize with septin rings (Fig. 3 F). Thus, it is possible that the geometry of the WAVE complex’s nanoscale rings may predict the WAVE complex’s preferred geometry. Whereas septins’ curved oligomerization and curvature preference have been shown for purified proteins, this has not been demonstrated for purified WAVE complex (WAVE complex does not oligomerize on its own; Chen et al., 2010). Future experiments will be needed to determine whether the geometry of oligomerization and/or other curvature-sensitive binding partners, and potentially in association with the WAVE complex’s activators, underlie the membrane curvature association of the WAVE complex.

### The WAVE complex plays a more central role than the ARP2/3 complex and IRSp53 in forming lamellipodia

As an actin NPF, the WAVE complex’s well-characterized link to the actin cytoskeleton is its activation of the Arp2/3 complex (Machesky and Insall, 1998; Pollard and Borisy, 2003; Weaver et al., 2003). Thus, it was surprising that the WAVE complex was not dependent on the Arp2/3 complex to build sheet-like protrusions. In the absence of ARP2, neutrophils still formed sheet-like protrusions with the WAVE complex and IRSp53 at their edges, whereas the formation of these protrusions required the WAVE complex; WAVE complex–null cells formed spiky protrusions (Fig. 7, A, B, and E). We also observed sheet-like protrusions in primary mouse bone marrow macrophages lacking the Arpc2 subunit (Fig. 7, C and D). Furthermore, WAVE2 without its Arp2/3 complex–interacting VCA domain also localized to the leading edge of sheet-like protrusions in HEK-293T cells (Fig. S5). These data suggest that the WAVE complex plays a central role in lamellipodial formation beyond its activation of the Arp2/3 complex, possibly reflecting WAVE’s polymerase activity. In addition to activating the nucleation of actin filaments through the Arp2/3 complex, WAVE can also accelerate the elongation of actin filaments by shuttling actin monomers onto the ends of uncapped actin filaments (Bieling et al., 2018). Actin polymerization is still required for these Arp2/3 complex–independent protrusions; F-actin is observed behind the WAVE crescents in these conditions (Fig. 7 F), and these extending protrusions are not observed in latrunculin-treated cells.

From our IRSp53 experiments, we found that IRSp53 requires its partnering interactions with the WAVE complex to enrich to lamellipodia and sites of saddle curvature, but IRSp53 is dispensable for the WAVE complex’s proper localization and patterning of lamellipodia (Fig. 6). Given that BAR domain proteins may have redundant functions (Chou et al., 2017; Tsujita et al., 2013; Insinna et al., 2019; Nkosi et al., 2013; Park et al., 2010), it is possible that other and/or a combination of I-BAR proteins are essential for proper WAVE complex localization. These data further highlight the central role of the WAVE complex in lamellipodial organization.

### Saddle curvature recognition of the WAVE complex may yield emergent behaviors, such as self-straightening lamellipodia and sealing of transcellular holes

The coupling of saddle curvature recognition (cell shape) and patterning F-actin polymerization (physical forces) could result in a number of emergent behaviors (Keren et al., 2008; Moreau et al., 2018). For example, if the WAVE complex both recognizes and generates sites of plasma membrane saddle curvature, this could form a feedback loop that organizes the expansion and self-straightening of lamellipodia. Near the initiation of a lamellipodium, a small sheet-like membrane deformation has saddle curvature at its lateral edges. WAVE complex association with these saddles, perhaps in a half ring–like form, would result in a laterally expanding zone of actin nucleation, where the wave would grow at the sides but be confined to a small region at the tip of the lamellipodium based on the negative curvature sensing. This spatial constraint on the positive feedback arm of the excitable actin nucleation circuit could explain why the domain of activation for this circuit is much thinner than other excitable circuits, such as cortical excitability in *Xenopus* (Bement et al., 2015) and Min protein oscillations for bacterial cell division (Loose et al., 2008).

Saddle enrichment would also enable maintenance of a uniform leading edge advancement. Any regions of the lamellipodia that lag behind also display saddle curvature, and the WAVE complex enrichment here would result in straightening of the front. Supporting this hypothesis, the WAVE complex preferentially enriches to flattening portions of closing transendothelial macroapertures (Fig. S3 C). Furthermore, recruitment of the WAVE complex could enable the recognition and actin-based sealing of saddle-shaped transendothelial macroaperture tunnels (Fig. 5; Maddugoda et al., 2011). Simple local rules of protein association could generate a number of the emergent features of cell shape and movement.

## Materials and methods

### Cell culture

All cells were cultured in a 37°C/5% CO_2_ incubator.

#### HL60s

HL60 cells (American Type Culture Collection [ATCC] CCL-240) were cultured in RPMI-1640 with 25 mM Hepes (Corning) with 10% (vol/vol) heat-inactivated FBS (Gibco) and maintained at 0.2–1.0 × 10^6^ cells/ml. Cells were differentiated with 1.5% DMSO (Sigma-Aldrich) in growth media for 4–5 d before experiments. All imaging was performed with dHL60s unless otherwise stated.

#### HEK-293Ts

HEK-293T cells (ATCC CRL-11268) were cultured in DMEM (Gibco) with 10% FBS. Cells were cultured up to 80% confluency.

#### HUVECs

HUVEC cells (ATCC CRL-1730) were a kind gift from the M.S. Conte laboratory (University of California, San Francisco [UCSF]). HUVECs were cultured in HyClone M-199 media (GE Healthcare) with 1% antibiotics-antimicrobial (Gibco), 15.3 units of heparin (Sigma-Aldrich), one vial of endothelial cell growth supplement (EMD Millipore), and 10% FBS. Cells were cultured up to 80% confluency, and growth media was replaced every other day. Experiments used cells with <10 passages.

#### B16F10

B16F10 cells (ATCC CRL-6475) were cultured in DMEM (Gibco) with 10% FBS, 1% penicillin-streptomycin (Gibco), and 1% GlutaMax (Life Technologies). Cells were cultured up to 80% confluency.

#### U2OS

U2OS cells (ATCC HTB-96) were a kind gift from the M. von Zastrow laboratory (UCSF). U2OS cells were cultured in McCoy’s 5A media (Gibco) with 10% FBS. Cells were cultured up to 80% confluency.

### Bone marrow–derived macrophages

Mouse primary bone marrow macrophage cells were a kind gift from the J.D. Rotty laboratory (Uniformed Services University of the Health Sciences, Bethesda, MD). Cells were cultured according to Rotty et al. (2017). Briefly, macrophages were cultured in 30% L-conditioned media and 70% DMEM with 10% FBS and 1% Glutamax. L-conditioned medium was generated by growing L929 cells until 80% confluency in DMEM with 10% FBS, then centrifuging and sterile-filtering the supernatant, which was kept in ∼45-ml aliquots at −80°C. To assess purity and maturity, macrophages were stained with two antibody cocktails (cocktail 1 for purity: CD11B, Ly6C, and CD47; cocktail 2 for maturation: CD11B, Ly6C, and F4/80; all at 1:100 in FACS buffer [1× PBS with 1% FBS]). For antibody staining, 2 × 10^5^ cells were pelleted and resuspended in antibody cocktail for 10 min at room temperature and then 30 min at 4°C, then washed and resuspended in FACS buffer and analyzed via flow cytometry during the first passage and 1 wk after culturing. To passage the macrophages, cells were washed with 1× PBS, incubated with 3 ml cold 0.5 mM EDTA at 4°C for 10 min, detached with a cell scraper, and then replated 1:2–1:4. To induce recombination and deletion of the *Arpc2* allele, cells at 50–70% confluency were treated with a first dose of 2 µM 4-OHT for 2 d then a second dose for 3 d. Cells were then plated for imaging and imaged the next day.

### Plasmids

Standard molecular biology protocols were followed for cloning. In general, DNA segments were PCR-amplified and cloned into a pHR lentiviral backbone (provided by the R.D. Vale laboratory, UCSF) with a spleen focus-forming virus promoter via standard Gibson assembly. Fluorescent proteins EGFP, mNeonGreen, mEos4b, mCherry, mRuby3, and mScarlet-I (all from the Michael Davidson laboratory collection, Florida State University, Tallahassee, FL) were PCR-amplified and cloned into a pHR plasmid containing Hem1 (Weiner et al., 2007; Diz-Muñoz et al., 2016). The other WAVE complex subunit constructs (EGFP-Abi1, EGFP-Abi2, EGFP-Sra1, EGFP-Nap1, and EGFP-WAVE2) were a kind gift from the G. Scita laboratory (European Institute of Oncology, Milan, Italy). IRSp53 was from the DNASU Plasmid Repository (ccsbBroadEn_11502) and subsequently subcloned into a pHR vector. IRSp53^1-250^ was “I-BAR,” the N-terminal 250 amino acids, and IRSp53^251-512^ was “C-term,” the C-terminal 262 amino acids beyond the I-BAR domain. For all IRSp53 constructs, the EGFP was C-terminally tagged.

### Cell line generation

#### Lentivirus

All HL60s, macrophages, and some HUVEC lines were stably expressing the constructs of interest. HEK-293Ts were plated in 6-well plates (Corning) until 70–80% confluency. Cells were transfected with 1.5 µg of the pHR plasmid along with two plasmids containing the lentiviral packaging proteins (0.167 µg of pMD2.G and 1.3 µg of p8.91) with Trans*IT*-293 (Mirus Bio). After 2–3 d of transfection, lentivirus-containing supernatant was collected, filtered with a 0.45-µm filter (EMD Millipore), and concentrated 20× with Lenti-X Concentrator (Takara). Lentivirus was used immediately or stored at −80°C. For HL60 transduction, 3.2 × 10^5^ cells, 4 µg/ml polybrene, and ∼130 µl of concentrated virus were incubated overnight. Cells were sorted via FACS (BD Aria2). For EGFP-tagged IRSp53 structure/function constructs, cells were sorted for comparable fluorescence expression levels (Fig. S4 C). For HUVEC transduction, a 50% confluent 6-well plate was incubated with 4 µg/ml polybrene and ∼130 µl of 1× virus overnight. For macrophage transduction, cells at 60–70% confluency were incubated with 4 µg/ml polybrene and 2× virus overnight.

#### Electroporation

For transient expression of plasmids in HUVEC cells, electroporation was used. For each electroporation condition, 5 × 10^4^ cells were resuspended in 10 µl of Buffer R (Thermo Fiser Scientific) with 500 ng of DNA. Electroporations were performed with the Neon Transfection System (Thermo Fisher Scientific) with 10-µl tips at 1,350 V for 30 ms for one pulse. Cells were recovered in 500 µl of culture media and imaged the next day.

#### Lipofectamine

For B16F10 and U2OS cell lines, plasmids were transiently expressed with Lipofectamine 2000 or 3000 (Invitrogen) per the manufacturer’s instructions.

#### Generation of CRISPR/Cas9 knock-in cell lines

Endogenous locus tagging with split-GFP or split-mNeonGreen is described in Leonetti et al. (2016). For each HL60 electroporation reaction, Cas9 RNP was generated by incubating 270 pmol of purified single-guide RNA (sgRNA) and 90 pmol of purified Cas9 at room temperature for 30 min and electroporated into cells with 100 pmol single-stranded homology-directed repair donor oligo. For each HEK-293T electroporation reaction, 50 pmol of RNP (with 65 pmol sgRNA) was electroporated with 120 pmol donor. For each HL60 reaction, 2 × 10^6^ cells expressing GFP1-10 (Addgene; #80409) or mNeonGreen1-10 (Addgene; #82610; induced via lentivirus transduction as described above) were resuspended in 100 µl of Buffer R with the RNP-donor mix and pulsed at 1,350 V for 35 ms for one pulse in Buffer E2 with the Neon Transfection System (Thermo Fisher Scientific). For each HEK-293T reaction, 1.65 × 10^5^ cells expressing GFP1-10 treated with 200 ng/ml nocodazole for 15–18 h were resuspended in 10 µl of Buffer R with the RNP-donor mix and pulsed at 1,150 V for 20 ms for two pulses in Buffer E with the Neon. Cells recovered in 20% FBS media. For full-length *WASF2* (WAVE2), the N terminus of exon 2 was targeted with sgRNA 5′-CCT​CGT​TAC​TAA​CGG​CAT​GG-3′ and repaired with donor 5′-GCT​TCT​AAC​GCT​AGG​CAA​CGT​CTG​ACG​GCA​CAG​GTG​CCT​TGG​CTC​GAT​GTT​CCT​CGT​TAC​TAA​CGG​GCC​GCC​CGA​ACC​GCC​GCC​ACC​TGT​AAT​CCC​AGC​AGC​ATT​TAC​ATA​CTC​ATG​AAG​GAC​CAT​GTG​GTC​ACG​CAT​GGT​GGA​CCT​GCT​TCA​GGC​AAT​GTT​CTG​AAT​GGT​GAA​AAA​CAA​CCT​AAA​AAA​GAA​ATA​ACA​GG-3′. To generate WAVE2ΔVCA cells, exon 8 was targeted with sgRNA 5′-GTC​GCT​ACG​GGC​ATC​GCT​CA-3′ and repaired with donor 5′-AAA​CCC​ACC​ATT​GAA​ATT​TAC​CTT​GAC​GGA​TGG​CTG​AAA​GCA​GGT​CGC​TAC​GGG​CAT​CGC​TTA​CTA​GCC​GCC​ACC​TGT​AAT​CCC​AGC​AGC​ATT​TAC​ATA​CTC​ATG​AAG​GAC​CAT​GTG​GTC​ACG​GCC​GCC​CGA​ACC​GCC​GCC​CGA​ACC​CAC​GGC​AGG​CAA​GGA​GGA​CTT​GGG​CTT​GGT​GGT​ATC​AGA​AAG​CGG​TGG-3′. For clathrin light chain A (*CLTA*), the reverse strand was targeted with sgRNA 5′-GAA​CGG​ATC​CAG​CTC​AGC​CA-3′ and repaired with donor 5′-CCA​GCG​CGG​GAC​CGC​CAG​GGG​CGC​CGG​CAG​GGG​CGC​CGA​ACG​GAT​CCA​GCT​CAG​CAC​CAC​TTC​CTG​GAC​CTT​GAA​ACA​AAA​CTT​CCA​ATC​CGC​CAC​CCA​TCA​TAT​CGG​TAA​AGG​CCT​TTT​GCC​ACT​CCT​TGA​AGT​TGA​GCT​CGG​TCA​TGG​CGG​GCA​ACT​GAA​CGG​CAC​GGA​CAC​CAA​CGG​TGA​GAC​AAA​AAC​CAA​CCG​AC-3′. After cells recovered, clonal cell lines were generated by FACS sorting EGFP- or mNeonGreen-positive cells into 96-well plates. Clones were screened via fluorescence microscopy and TOPO TA sequencing (Invitrogen). For HEK-293T clonal lines, an ∼400 base pair sequence spanning the guide targeting the VCA region was PCR amplified with primers containing GENEWIZ Illumina adaptor sequences and gel extracted, and MiSeq was performed using the GENEWIZ Amplicon-EZ service. Resulting paired-end reads were analyzed for editing using CRISPResso2 (Clement et al., 2019).

#### Generation of CRISPR/Cas9 knockout cell lines

Wild type HL60 cells were first transduced with a plasmid expressing a human codon optimized *Streptococcus pyrogenes* Cas9 with a C-terminal tagBFP fusion (gift from S. Qi, Stanford University, Stanford, CA) where high tagBFP-expressing cells were sorted via FACS. Cas9-expressing cells were then transduced with a lentiGuide-Puro plasmid (Addgene; #52963; Sanjana et al., 2014) containing a puromycin-selectable gRNA targeting exon 2 of IRSp53/*BAIAP2* (5′-TCC​GGA​ACT​TCA​TCG​CCA​TG-3′). After 1 wk of puromycin selection, clonal cell lines were generated by diluting cells into 96-well plates at a 0.5 cell/well density. Clonal lines were verified by genomic DNA sequencing of the region flanking the Cas9 cut site, TOPO TA cloning (Invitrogen), and immunoblotting. WAVE complex-null and ARP2-null HL60 cells were from Graziano et al. (2019).

#### Immunoblotting

For primary antibodies IRSp53 (rabbit; 1:100; Abcam; #ab37542), β-actin (mouse; 1:1,000; Cell Signaling Technology; #3700), WAVE2 (rabbit; 1:1,000; Cell Signaling Technology; #3659), Arp2 (rabbit; 1:1,000; GeneTex; #GTX103311), and GAPDH (mouse; 1:2,000; Invitrogen; #MA5-15738), protein samples from 10^6^ cells were prepared in 2× Laemmli sample buffer, subjected to a SDS-PAGE gel with MES running buffer, and transferred to a nitrocellulose membrane. Membranes were blocked at room temperature for 30–60 min in a 1:1 solution of TBS (20 mM Tris, 500 mM NaCl, pH 7.4) and Odyssey Blocking Buffer (LI-COR) and then incubated overnight at 4°C with diluted primary antibodies in a solution of 1:1 TBS with 0.2% wt/vol Tween-20 (TBS-T) and Odyssey Blocking Buffer. Membranes were washed three times with TBS-T and then incubated for 1 h at room temperature with secondary antibodies diluted 1:20,000 in Odyssey Blocking Buffer. Secondary antibodies used were IRDye 680RD goat anti-mouse (LI-COR) and IRDye 800CW goat anti-rabbit (LI-COR). Membranes were washed three times with TBS-T and one time with TBS.

For primary antibodies HEM1 (rabbit; 0.4 µg/ml; Novus Biologicals; #13643) and GAPDH (mouse; 1:2,000; Invitrogen; #MA5-15738), 10 × 10^6^ cells/ml samples were lysed in ice-cold 2× lysis buffer (1× lysis buffer: 25 mM Tris-HCl, 150 mM NaCl, 5 mM MgCl_2_, 1% NP-40, 1 mM DTT, 5% glycerol, 1 mM PMSF, and cOmplete protease inhibitor cocktail [Roche]) and spun at 21,000 × *g* for 20 min at 4°C. Supernatant was removed and prepared in 2× Laemmli sample buffer, then subjected to a SDS-PAGE gel with MOPS running buffer and transferred to a polyvinylidene difluoride (PVDF) membrane. Membranes were blocked for 30–60 min in a 1:1 TBS:Odyssey Blocking Buffer solution and then incubated overnight at 4°C with primary antibodies diluted in a solution of 1:1 TBS-T:Odyssey Blocking Buffer. Membranes were washed three times with TBS-T and then incubated for 1 h at room temperature with secondary antibodies diluted 1:10,000 in Odyssey Blocking Buffer. Secondary HRP-conjugated antibodies used were goat anti-mouse IgG (Invitrogen; #62-6520) and goat anti-rabbit IgG (Invitrogen; #65-6120). Membranes were washed three times with TBS-T and one time with TBS. The membranes were treated with a SuperSignal West Femto kit (Thermo Fisher Scientific), following the manufacturer’s instructions, immediately before imaging.

For primary antibodies Arpc2 (rabbit; 1:500; EMD Millipore; 07–227) and GAPDH (mouse; 1:1,000; Invitrogen; #MA5-15738), sample lysates were prepared with ice-cold RIPA buffer with protease (cOmplete protease inhibitor cocktail; Roche) and phosphatase (Phosphatase Inhibitor Mini Tablets; Pierce) inhibitors, rotated for 15 min at 4°C, and spun at 21,000 × *g* for 10 min at 4°C. ECL Western blots were performed as described above except for a 1:1 solution of PBS-Tween (0.2%) and 5% milk solution for block and antibodies incubation steps.

All blots were imaged with an Odyssey Fc (LI-COR) and analyzed with ImageStudioLite.

### Immunostaining

Cells were fixed as described and blocked with 3% BSA and 0.1% Triton X-100 diluted in PBS for 1 h at room temperature. Primary antibody was diluted in blocking solution at 4°C overnight, washed three times with PBS, and then incubated with the secondary antibody and washed three times with PBS. SEPT7 (rabbit; IBL; #18991) primary antibody was used at 1:1,000 dilution with secondary antibody goat anti-rabbit IgG conjugated to Alexa Fluor 680 (Invitrogen; #A-21076) at 1:1,000 dilution.

### Imaging

#### Cell preparation

##### HL60s

For imaging, differentiated cells were resuspended in imaging media (either Leibovitz’s L-15 [Gibco] with 0.5% FBS or modified HBSS [10× stock consists of 1,500 mM NaCl, 40 mM KCl, 10 mM MgCl_2_, 12 mM CaCl_2_, 100 mM glucose, and 200 mM Hepes, pH 7.2]). Cells were plated onto fibronectin-coated wells (100 µg/ml for 1 h at room temperature) and incubated (37°C/5% CO_2_) for at least 7 min before two or three washes with imaging media. For chemoattractant stimulation, a 2× stock of 20 nM N-formyl-L-methionyl-L-leucyl-L-phenylalanine (fMLP; Sigma-Aldrich) was added. For additional chemoattractant stimulation, a 2× stock of 200 nM fMLP was added. For F-actin inhibition, a 2× stock of 1 µM latrunculin B (EMD Millipore and Sigma-Aldrich) with 200 nM PMA (Sigma-Aldrich; for persistent Hem1 activation) was used, unless noted otherwise. Other F-actin inhibition drugs were latrunculin A (EMD Millipore) and cytochalasin B (Sigma-Aldrich). All initial stocks were dissolved in 100% dry DMSO and freshly diluted in imaging media before experiments. *C. difficile* toxin B (Sigma-Aldrich) was added to the cells at a final concentration of 1 µg/ml for at least 4 h.

##### HUVECs

To induce TEM formation, HUVECs were incubated in 50 µM Y27632 (Tocris and Sigma-Aldrich) for at least 4 h before imaging. Y27632 was present throughout imaging.

##### Membrane labeling

For membrane labeling, Vybrant DiD (Invitrogen) or CellMask Deep Red (Invitrogen) was freshly diluted (50–500 nM) in imaging media. HL60s were labeled in suspension for 30 s at 37°C and washed two or three times with imaging media. Adherent cell lines were labeled for 5 min at 37°C and washed three to five times with PBS.

##### Fixation

Cells were plated in #1.5, 8-well Lab-Tek II chambers (Thermo Fisher Scientific). To fix cells, medium was aspirated while simultaneously adding 200 µl of 2% glutaraldehyde (Sigma-Aldrich) for 10 min and washed with PBS for 30 s and 60 s. Aldehydes were quenched with 0.1% sodium borohydride (Sigma-Aldrich) for 7 min followed by two or three 10-min PBS washes. Samples were imaged immediately or stored at 4°C. All dilutions were prepared fresh in cytoskeleton buffer with sucrose (CBS), a T.J. Mitchison laboratory (Harvard Medical School, Boston, MA) recipe: 10 mM MES, pH 6.1, 138 mM KCl, 3 mM MgCl, and 2 mM EGTA, with 0.32 M sucrose added fresh before use. HUVECs were fixed with 4% PFA in PBS, pH 7.4. All materials were warmed to 37°C.

##### Phalloidin staining

Cells were fixed as described above and stained with Alexa Fluor 647 phalloidin (5 µl/ml; Invitrogen) in 1× CBS buffer for 15–45 min and washed with CBS buffer. Samples were imaged immediately or stored at 4°C.

#### Microscopes

The imaging media are described above. The fluorescent protein or fluorochrome imaged is described in the figure and/or figure legend.

##### TIRF-SIM

TIRF-SIM imaging was performed on a DeltaVision OMX SR (GE Healthcare) equipped with three PCO 15-bit CMOS cameras with a 60×/1.42 NA Plan Apo objective (Olympus) with 1.516 or 1.518 refractive index oil (Cargille). HL60 experiments were performed at room temperature while adherent cells were performed at 37°C/5% CO_2_. Images were acquired with Acquire SR software and processed with softWoRx. SIM images were reconstructed using OMX SI Reconstruction with the default parameters including Wiener filter: 0.001 and multichannel images were aligned using OMX Align Images with the Legacy Image Registration method. Display of some TIRF-SIM membrane images were smoothed in Fiji. To generate conventional resolution TIRF images, the nine images (three phases × three angles) that construct the TIRF-SIM images were sum-projected.

##### Spinning-disk confocal

Images were acquired on a Nikon Eclipse Ti microscope with a 60×/1.40 NA Plan Apo objective (Nikon), Yokogawa CSU-X1 spinning-disk confocal, and a Prime 95B cMOS camera (Photometrics). 405-, 488-, 561-, and 640-nm laser lines (Agilent Technologies) and environmental control (37°C/5% CO_2_; Okolab) were used. Software was controlled with Nikon Elements.

##### TIRF

All TIRF images except for Fig. S5 C were acquired with the DeltaVision OMX SR (GE Healthcare) equipped with three PCO 15-bit CMOS cameras with a 60×/1.42 NA Plan Apo objective (Olympus) with 1.516 or 1.518 refractive index oil (Cargille) in ring-TIRF mode with either sequential or simultaneous excitation. Images were acquired with Acquire SR and deconvolved (default settings) via softWoRx. TIRF images in Fig. S5 C were acquired on a Nikon Eclipse Ti microscope equipped with a Borealis beam-condition unit (Andor Technology), a 60×/1.49 NA Plan Apo TIRF objective (Nikon), and an iXon Ultra EMCCD camera. Environmental control (37°C/5% CO_2_; Okolab) was used. Acquisition was controlled with Micro-Manager (UCSF; Edelstein et al., 2014).

##### Lattice light sheet and processing

Lattice-light sheet imaging was performed on a home-built microscope as designed and in a manner previously described (Chen et al., 2014) and followed an established protocol (Cai et al., 2017). Briefly, 5-mm round coverslips (Warner Instruments) were plasma cleaned and fibronectin coated (100 µg/ml for 1 h). Cells were plated as described above. The coverslip sample was then loaded into a sample holder and placed into the previously conditioned microscope sample bath (37°C with 25 nM fMLP chemoattractant) and secured. Imaging was performed with a 25×/1.1 NA Apo LWD 2.0 objective, a 488-nm laser (MPBC), and a Hamamatsu Orca Flash 4.0 camera. Camera exposure was 10 ms per frame, leading to a temporal resolution of 2.25 s in single-color mode. Acquisition software was written in LabView by the E. Betzig laboratory (Janelia Research Campus, Ashburn, VA).

Raw image files were deconvolved using the iterative Richardson–Lucy algorithm with the known point spread function for each channel and were collected before each day of imaging (Chen et al., 2014). The code for this process was provided by the E. Betzig laboratory originally written in MATLAB (MathWorks) and ported into CUDA (Nvidia) for parallel processing on the graphics processing unit (Nvidia GeForce GTX Titan X). Each sample area underwent 15 iterations of deconvolution.

Regions of interest within the sampling were cropped down to size and compressed from 32-bit TIFFs to 16-bit TIFFs using in-house MATLAB code to allow immigration into the 3D visualization software ChimeraX (UCSF, Resource for Biocomputing, Visualization, and Informatics; Goddard et al., 2018). To highlight intensity ranges, additional channels were created by thresholding.

##### Transmission electron microscopy

Cells were prepared for conventional electron microscopy in two ways: cells were either plated on fibronectin-coated ACLAR discs (TedPella), fixed with glutaraldehyde as described above, and dehydrated and embedded in resin; or cells were plated on sapphire disks and fixed via high-pressure freezing/freeze-substitution. For the glutaraldehyde fixation method, cells were treated with 100 nM chemoattractant or 500 nM latrunculin B for 5 min, fixed, stained with uranyl acetate and OsO_4_, dehydrated with cold ethanol, and embedded with Epon 812 resin. For the high-pressure freezing/freeze-substitution method, cells were plated onto 3-mm-diameter sapphire disks and treated with 100 nM chemoattractant or 500 nM latrunculin B for 5 min before freezing. The sapphire disk was then placed, cells toward the inside of 100-µm-well specimen carriers (Type A; Technotrade International Inc.) containing 20% BSA in growth media. The sandwiched cells were frozen using a BalTec HPM 01 high-pressure freezer (BalTec). Freeze-substitution in 1% OsO_4_, 0.1% uranyl acetate, and 1% methanol in acetone, containing 3% water (Buser and Walther, 2008; Walther and Ziegler, 2002), was performed with a Leica AFS2 unit. Following substitution, samples were rinsed in acetone, infiltrated, and then polymerized in Eponate 12 resin (Ted Pella). For conventional electron microscopy, serial 50-nm sections were cut with a Leica UCT ultramicrotome using a Diatome diamond knife, picked up on Pioloform-coated slot grids, and stained with uranyl acetate and Sato’s lead (Sato, 1968). Sections were imaged with a FEI Tecnai T12 TEM at 120 kV using a Gatan 2k × 2k camera. For electron microscopy tomography, 200-nm sections were cut, mounted on grids, and stained as above for serial thin sections. Tomograms were acquired using an FEI T20 TEM at 200 kV, and tomograms reconstructed using eTomo/IMOD (Kremer et al., 1996; Mastronarde, 1997).

##### Airyscan

Images were acquired on a Zeiss laser scanning microscope (LSM) 800 or 880 with Airyscan with a 63×/1.40 NA Plan Apo DIC M27 objective (Zeiss), Airyscan (GaAsP PMT) detector, and environmental control (37°C/5% CO_2_). Zen Blue was used to acquire images and perform Airyscan processing (default settings).

##### Stimulated emission depletion

Images were acquired on a Leica TCS SP8 X Confocal microscope with a 100×/1.40 NA HC Plan Apo objective, GaAsP Hybrid detectors, tunable 470–670-nm white light laser system, and environmental control (37°C/5% CO_2_). Software was controlled with Leica Application Suite X, and images underwent Huygens deconvolution.

##### Nikon-SIM

Images were acquired on a Nikon Ti microscope with N-SIM with a 100×/1.49 NA Apo TIRF SR objective (Nikon) and Andor DU-897 camera at room temperature. Nikon Elements was used to control acquisition and reconstruct images (default parameters).

##### Photoactivated localization microscopy

Images were acquired similarly to Pessino et al. (2017). Briefly, images were acquired with the B. Huang laboratory (UCSF) home-built stochastic optical reconstruction microscope based on a Nikon Eclipse Ti-U microscope at room temperature with a 100×/1.4 NA UPlanSApo objective (Olympus), iXon+ DU897E-C20-BV camera (Andor), and 405- and 561-nm lasers (OBIS). Home-written software was used to acquire images, and Insight3 software was used to reconstruct images.

### Image analysis and statistics

All image analysis was performed in Fiji and/or Python. Data handling and statistical tests were performed with Python and Prism 7–8 (GraphPad).

#### Ring identification and diameter calculation

After background subtraction (rolling ball), segmentation was performed with Trainable Weka Segmentation (Arganda-Carreras et al., 2017) machine-learning algorithm to identify ring structures. The diameter of a perfect circle with the same perimeter of the ring particle was calculated (diameter = C/*π*; C, circumference). Fig. 2 D displays a histogram of each individual ring’s diameter, while Fig. S2 B displays the interquartile range of ring diameters across F-actin inhibition conditions. The number of rings identified in the segmentation across conditions was graphed, and two-tailed, unpaired *t* tests were performed.

#### Membrane curvature and velocity

Fluorescence images were segmented using a three-step process consisting of Gaussian smoothing, intensity-based thresholding, and binary erosion. The threshold and degree of erosion were chosen manually to align the boundary of the binary image with the apparent edge of the cell membrane. To facilitate temporal analysis of edge properties, these boundaries were then fit using a spline interpolation consisting of 1,000 points. Edge velocity at a particular point, P, at time, t, was estimated by calculating the average of the distance transforms of the binary images at times t−1 and t+1 and interpolating the value of this function at the coordinates of P. The radius of curvature at a point, P, was calculated by approximating the radius of the osculating circle, C, along the boundary at the coordinates of P. To approximate this osculating circle, we chose a scale parameter, S, then collected the coordinates of the two points at indices S units away from P. To adjust for the image pixel size, S = 10 for TEM images, and S = 44 for the HL60 migrating cell. Given these three points, we calculated the unique equation of a circle passing through them. A sign could then be assigned by comparing the vector between P and the center of circle C and the normal vector of the boundary.

To calculate WAVE complex signal intensity, we expanded the boundary in both directions to create a ring-shaped region and calculated the average background-corrected signal as a function of angle relative to the center of the region. For the TEM data, we calculated the binned average fluorescence signals from 0–360° in 7.2° increments (for 50 bins total). For the HL60 migrating cell dataset, 1,000 bins were used. Normalized WAVE complex intensity is the ratio of background-corrected WAVE signal to the background-corrected membrane signal (which was calculated similarly). To standardize the edge measurements across time and across TEMs, and to make comparisons between geometric and fluorescence features, we employed a similar binning strategy for the geometric features. Spearman’s correlation coefficient was calculated in Fig. S3, A and B.

Based on simulations that incorporated expected WAVE complex behaviors (for example, residence time on the membrane), we expected that an instantaneous statistical relationship between WAVE signal and curvature would be difficult to detect in a system with an intact cytoskeleton. Thus, we looked for enrichment of WAVE signal in areas of positive curvature that were becoming less positive (flattening) or becoming more positive (lagging). By calculating the change in angle-binned average curvature over time, we isolated regions with positive curvature that had a positive derivative and regions with positive curvature that had a negative derivative. We then extracted and compared the fluorescence values from these regions. By artificially rotating the normalized WAVE fluorescence values by one increment (7.2°) and recalculating the enrichment scores, we can estimate the significance of the enrichment of WAVE signal in flattening areas over lagging areas. Enrichment scores were compared with the null value of 1 (no preference between flattening and lagging) using a two-tailed, unpaired *t* test at each rotation value in Fig. S3 C. Analysis can be found at https://github.com/weinerlab/wavecomplex_tem_analysis.

####  Membrane curvature of electron micrographs

After identifying the plasma membrane, a perfect circle was fitted to both sides of the neck of an invagination and at the tip of a protrusion. Curvature, *κ*, was defined as *κ* = 1/r (r, radius). A two-tailed, unpaired *t* test was performed.

#### TEM enrichment

TEMs were identified by segmenting the membrane channel (TEM mask). The signal mask was generated by dilating the TEM mask by 2–3 pixels and the background mask was generated by dilating the signal mask by 2–3 pixels. A doughnut-shaped signal segment was calculated by subtracting the TEM mask from the signal mask, and a doughnut-shaped background segment was calculated by subtracting the signal mask from the background mask. The doughnut-shaped signal segment was background-corrected (subtracting the background intensity per unit area multiplied by the signal mask area). The enrichment factor was measured as the ratio of the signal to background intensities per unit areas. The intensity per unit perimeter was calculated as the background-corrected signal intensity divided by the signal perimeter. Spearman’s correlation coefficients for the WAVE complex and membrane for each TEM throughout closure were calculated, and a two-tailed, unpaired *t* test comparing the Spearman’s coefficients was performed in Fig. 5 E. A two-tailed Mann–Whitney test was performed in Fig. S4 D.

#### IRSp53 and Hem1 colocalization

Images were background subtracted (rolling ball), and Fiji’s coloc2 function was applied with the segmented images and a region of interest of the combined masks.

#### IRSp53 lamellipodia/filopodia enrichment

After background subtraction (rolling ball), two masks were generated: a lamellipodia mask was created from Hem1 signal, and a filopodia mask was created from the eroded regions of a cell mask. After subtracting the cytosolic background from each segment (cytosolic intensity per unit area multiplied by the segment’s area), the intensity per unit area of each segment was calculated, i.e., lamellipodia signal/lamellipodia area. The ratio of the lamellipodia to the filopodia intensities per unit areas was graphed. A two-tailed, unpaired *t* test was performed.

#### IRSp53 structure/function ring enrichment

After background subtraction (rolling ball), a ring mask was generated from segmenting Hem1 signal, and a background mask was generated by dilating the ring mask. IRSp53 signal was measured from both masks. After background correction (subtracting the background intensity per unit area multiplied by the ring mask area), the enrichment factor was calculated as the ratio of the ring to the background intensities per unit areas. Enrichment of all rings within a cell was averaged and treated as an individual data point. A two-tailed, one-way ANOVA with Tukey’s multiple comparisons follow-up tests was performed.

#### Phalloidin intensity

After background subtraction (rolling ball), a Hem1 mask was generated from segmenting Hem1 intensity signal. The phalloidin signal within the Hem1 mask was compared with the signal within the same mask of a randomized version of the phalloidin image. Two-tailed, unpaired *t* tests were performed.

#### Protrusion analysis

Fluorescence images were segmented with Gaussian smoothing, intensity-based thresholding, and binary erosion. Manual drift correction was applied as needed. To identify protrusions, difference maps (of 1-min intervals) were generated by subtracting the segmented masks. For protrusion widths, the width at the midpoint of the extension’s length was measured. For protrusion speed, the extension’s length over 1 min was measured. Kruskal–Wallis tests with Dunn’s multiple comparisons follow-up tests were performed in Fig. 7 B, and Mann–Whitney tests were performed in Fig. 7 D.

### Online supplemental material

Fig. S1 shows Western blots of HEM1 and WAVE2 expression levels from the Hem1-EGFP HL60 cell line used throughout the study, data from endogenously split-GFP–labeled WAVE2, and phalloidin staining. Fig. S2 shows the WAVE complex rings across different actin polymerization inhibitors and concentrations, multiple super-resolution microscopy techniques, tagged fluorescent protein, labeled WAVE complex subunits, and cell lines. Fig. S3 shows plots comparing WAVE complex signal and membrane velocity as well as its enrichment in flattening versus lagging membrane regions. Fig. S4 shows additional IRSp53 data: TIRF-SIM images of high-expression of IRSp53, IRSp53 ring measurements, flow cytometry plots of IRSp53 structure/function constructs, IRSp53 structure/function confocal imaging in HUVECs, and characterization of the CRISPR/Cas9 knockout of IRSp53. Fig. S5 shows the generation of the WAVE2ΔVCA split-GFP endogenous knock-in HEK-293T cell line and its localization to the leading edge of sheet-like protrusions. Video 1 shows lattice light sheet and TIRF imaging of the WAVE complex at protruding lamellipodia. Video 2 shows TIRF-SIM versus sum-projected TIRF-SIM imaging of WAVE complex rings in the absence of actin polymer. Video 3 shows TIRF-SIM imaging of WAVE complex rings at the necks of plasma membrane invaginations. Video 4 shows WAVE complex and IRSp53 at TEMs. Video 5 shows TIRF-SIM imaging of IRSp53 and WAVE complex colocalization at a protruding lamellipodium. Video 6 shows TIRF-SIM imaging of the induction of IRSp53 ring structures in the presence of F-actin. Video 7 shows sheet-like protrusions in ARP2-disrupted dHL60 cells. Video 8 shows sheet-like protrusions in *Arpc2*^−/−^ macrophage cells. Video 9 shows the WAVE complex localization in ARP2-disrupted dHL60 cells. Video 10 shows that WAVE2ΔVCA localizes to the leading edge of sheet-like protrusions.
